# Bias in the arrival of variation can dominate over natural selection in Richard Dawkins’s biomorphs

**DOI:** 10.1371/journal.pcbi.1011893

**Published:** 2024-03-27

**Authors:** Nora S. Martin, Chico Q. Camargo, Ard A. Louis

**Affiliations:** 1 Rudolf Peierls Centre for Theoretical Physics, University of Oxford, Oxford, United Kingdom; 2 College of Engineering, Mathematics and Physical Sciences, University of Exeter, Exeter, United Kingdom; Universite de Lausanne Faculte de biologie et medecine, SWITZERLAND

## Abstract

Biomorphs, Richard Dawkins’s iconic model of morphological evolution, are traditionally used to demonstrate the power of natural selection to generate biological order from random mutations. Here we show that biomorphs can also be used to illustrate how developmental bias shapes adaptive evolutionary outcomes. In particular, we find that biomorphs exhibit phenotype bias, a type of developmental bias where certain phenotypes can be many orders of magnitude more likely than others to appear through random mutations. Moreover, this bias exhibits a strong preference for simpler phenotypes with low descriptional complexity. Such bias towards simplicity is formalised by an information-theoretic principle that can be intuitively understood from a picture of evolution randomly searching in the space of algorithms. By using population genetics simulations, we demonstrate how moderately adaptive phenotypic variation that appears more frequently upon random mutations can fix at the expense of more highly adaptive biomorph phenotypes that are less frequent. This result, as well as many other patterns found in the structure of variation for the biomorphs, such as high mutational robustness and a positive correlation between phenotype evolvability and robustness, closely resemble findings in molecular genotype-phenotype maps. Many of these patterns can be explained with an analytic model based on constrained and unconstrained sections of the genome. We postulate that the phenotype bias towards simplicity and other patterns biomorphs share with molecular genotype-phenotype maps may hold more widely for developmental systems.

## Author summary

The question of how novel phenotypic variation arises has received much less attention than natural selection even though both are key processes in Darwinian evolution. Here we uncover striking anisotropic structure in phenotypic variation for biomorphs, Richard Dawkins’s iconic schematic model of development, introduced in his book *The Blind Watchmaker*. We observe strong simplicity bias: upon uniform random sampling of genotypes, a simple biomorph can be exponentially more likely to appear as potential variation than any individual complex biomorph is. The mapping from genotypes to phenotypes (GP map) for biomorphs exhibits many similarities to molecular GP maps, including relatively high mutational phenotype robustness which is positively correlated with evolvability. Such structure in the arrival of variation can enhance the ability of evolution to find fitness maxima through neutral exploration. By using evolutionary simulations, we show that phenotypes that fix in a population may not be the fittest ones, but rather adaptive phenotypes that are most likely to appear as variation. We hypothesize that similar developmental bias as observed in the biomorphs system may occur more widely in developmental systems.

## Introduction

### Three versions of the infinite monkey theorem

**Fig 1:**
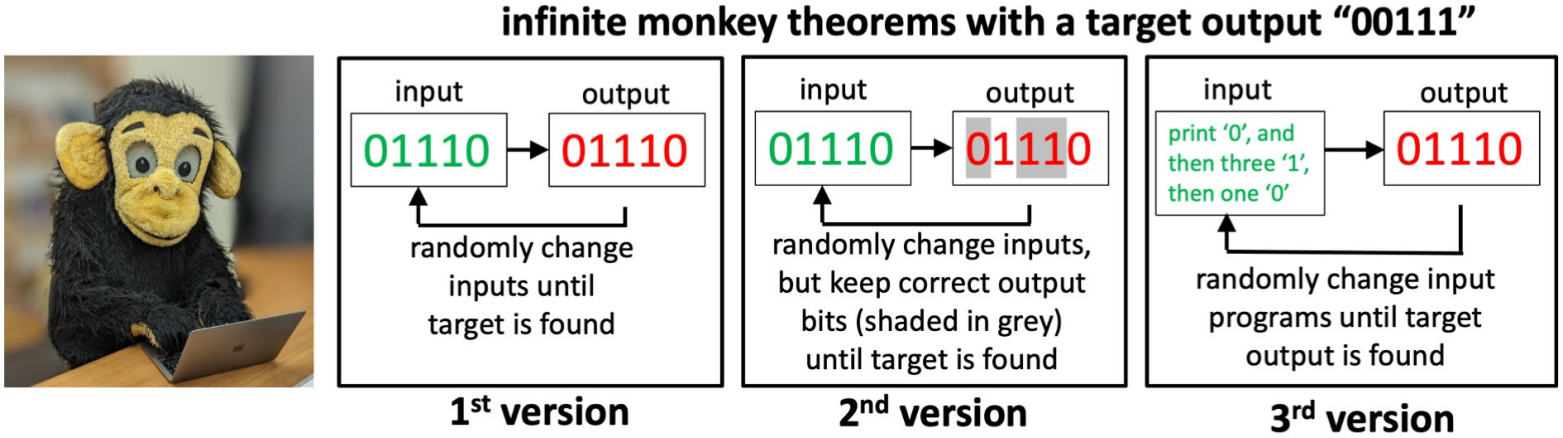
Three versions of the “infinite monkey theorem” compared. In the 1^st^ version, or the classic case, all outputs of length *n* are equally likely, and thus the probability of obtaining a specific output scales as 1/*M*^*n*^, where *M* is the number of keys, and *n* is the length of the desired output. In the 2^nd^ version, introduced by Dawkins’s [1], a fitness function fixes each correct partial output, so that a desired *n*-length string is likely to be found in a timescale that scales as *n* × *M*, which is linear instead of exponential in *n*. In the 3^rd^ algorithmic version, the probability of obtaining an output scales as 1/*M*^*K*^, where *K* is the length of a program that generates it [3]. Outputs for which short programs exist therefore appear with exponentially higher probability. The length of the shortest program with which a given output can be produced is related to the famous Kolmogorov complexity measure [4] so that the algorithmic monkey theorem also implies a bias towards simplicity.

In his influential book, *The Blind Watchmaker* [1], Richard Dawkins’s illustrates how natural selection can efficiently find fitness maxima in ‘hyper-astronomically large’ [2] search spaces by introducing an intriguing twist on the famous infinite monkey theorem. He frames his argument by first introducing the classic case (see Fig 1) with a question: How likely is it that a monkey randomly typing on a typewriter produces Hamlet’s 28-character phrase “METHINKS IT IS LIKE A WEASEL”? For a monkey typing on an *M*-key typewriter, the probability to produce a specific string of *n* characters will scale as 1/*M*^*n*^, which rapidly becomes unimaginably small with increasing *n*. By analogy, random mutations on their own are unlikely to produce meaningful biological novelty. Dawkins’s contrasts this picture with his second version of the infinite monkey theorem, where a fitness function acts on each letter independently. The output stops changing once the correct letter is found, so that on average only *M* random keystrokes are needed for each letter. Thus, any *n* letter phrase can be produced in a number of keystrokes that scales as *n* × *M*, which is exponentially smaller than in the first case. This simple but evocative example illustrates an important property of biological sequence spaces. For a given alphabet size *M*, their size grows exponentially with sequence length *L* as *M*^*L*^, but genomic distances remain linear in *L* because on the order of *L* mutations can be used to link any two sequences. By using fitness functions of the kind that Dawkins’s introduced, an evolutionary search algorithm can exploit this linearity and locate a fitness maximum in an exponentially large high-dimensional search space within a relatively small number of randomly generated steps.

In this paper, we explore the evolutionary consequences of a third (algorithmic) version of this famous trope of monkeys on keyboards (see Fig 1). In Dawkins’s version, the monkeys directly type out components of the outputs, i.e. the phenotypes. In evolution, however, novel phenotypic variation is generated indirectly by random mutations which are then “decoded” through the process of development. To capture this mapping from genotypes (the inputs) to phenotypes (the outputs), consider instead monkeys generating outputs by typing at random into a computer programming language [3]. In contrast to the classical version of the infinite monkey theorem, where all output strings of length *n* are equally likely (with probability *p* = 1/*M*^*n*^), in the algorithmic picture, certain outputs appear exponentially more frequently than others. Consider the following example (from ref [3]): a string of length *n* = 1000 of the form “010101…” would appear when typing the 21-character program “print ‘01’ 500 times;”. Therefore, its probability *p* = 1/*M*^21^ is many orders of magnitude larger than the probability *p* = 1/*M*^1000^ for the classical version. Thus, within this algorithmic picture, there are certain kinds of outputs, namely those for which short programs exist, which have an exponentially higher probability than outputs without such short algorithmic descriptions [3]. Interestingly, an algorithmic picture of evolution is also introduced in a famous passage from chapter 5 of *the Blind Watchmaker* [1], where Dawkins’s describes seeds falling from a tree: *“It is raining instructions out there; it’s raining programs; it’s raining tree-growing, fluff-spreading, algorithms. That is not a metaphor, it is the plain truth. It couldn’t be any plainer if it were raining floppy discs.”*.

### Formalising the algorithmic infinite monkey theorem with algorithmic information theory (AIT)

Can the intuitive link between our simple algorithmic picture and the mapping from genotypes to phenotypes be made more rigorous? To this end, we turn to the field of algorithmic information theory (AIT) [4] where a central concept is the algorithmic probability *P*(*x*) that a universal Turing machine, a computing device that can perform any possible computation, generates a particular output *x* upon random sampling of input programs. This probability decays exponentially with the Kolmogorov complexity *K*(*x*) of the output string *x*, where *K*(*x*) is the length of the shortest program with which *x* can be produced on the universal Turing machine. Because *K*(*x*) is the length of the shortest program that generates output *x*, these concepts from AIT formalise the algorithmic picture of monkeys typing into a computer programming language: The shortest program has the highest probability. One difficulty with these formal arguments is that many input-output maps where one might want to apply the intuition of the monkeys on keyboards are not the universal Turing machines upon which AIT relies. However, an upper bound has recently been derived for the probability *P*(*x*) that an output *x* is obtained upon random sampling of inputs for a broad class of computable input-output maps [5]. It takes the specific form:

$$\begin{array}{c} P\left(x\right)\leq 2^{-a\tilde{K}\left(x\right)+b}, \end{array}$$ (1)

where the descriptional complexity $\tilde{K}\left(x\right)$ is a suitable approximation to the (uncomputable) Kolmogorov complexity, and two constants *a* and *b* are independent of the outputs *x*. Typically $\tilde{K}\left(x\right)$ is based on some measure of compression [5]. This relationship between probability and the complexity of the output has been called “simplicity bias” in the context of computable input-output maps [5]: outputs with high *P*(*x*) will have small $\tilde{K}\left(x\right)$, and outputs with large $\tilde{K}\left(x\right)$ will have low *P*(*x*) (but not necessarily vice-versa because Eq 1 is an upper bound). In [3, 5, 6] it was shown that this bound holds for a wide range of input-output maps.

### Simplicity bias in genotype-phenotype maps

It has recently been argued [3] that many genotype-to-phenotype (GP) maps obey the mathematical conditions needed for Eq 1 to be satisfied, formalizing the intuitive connection between GP maps and the algorithmic infinite monkey theorem.

GP maps typically exhibit redundancy due to neutral mutations [7], where ‘neutral’ simply means that the mutation does not change the phenotype, which is a simpler definition than the classical notion introduced by Kimura [8]. This redundancy naturally leads to the concept of a *neutral set* made up of all the genotypes that map to a given phenotype *p*. We can define the associated probability *P*(*p*) that a randomly selected genotype belongs to the neutral set of *p*, which is also referred to as the phenotype frequency *f*_*p*_ of *p*. It is directly proportional to the size of the neutral set. *Phenotype bias* occurs when there are large differences in the *neutral set sizes* (or equivalently in the *f*_*p*_) associated with different phenotypes *p* [9].

Strong evidence for this “simplicity bias” was found at the molecular scale for the GP maps of RNA secondary structure, the polyomino model for protein quaternary structure, and a popular model of the yeast cell-cycle gene regulatory network [3]. For example, phenotype bias towards simplicity can explain key patterns in nature such as an observed strong preference for symmetry in protein complexes, and the fact that the most frequent RNA secondary structures found in nature have structures that are highly compressible, and therefore are simple with low descriptional complexity $\tilde{K}\left(p\right)$ [3]. In RNA especially, detailed quantitative comparisons are possible: For example, if the secondary structures are coarse-grained using level-5 of the RNAshapes method [10], then the 68 evolved secondary structures of length *L* = 126 found in the RNAcentral database [11] of functional RNA are among the 96 structures with highest phenotypic frequencies out of a much larger set of 10^12^ topologically possible level 5 structures [9]. This observation does not negate the role of selection. Each functional RNA structure in the database will have fixed due to natural selection, and a randomly selected sequence would be unlikely to perform a given biological function (see [12] for a recent discussion). But it does mean that nature was able to produce the “*endless forms most beautiful*” [13] of the living world from only a minuscule fraction of the set of all RNA structures, namely those that are most likely to appear as variation.

The mechanisms by which strong phenotype bias is predicted to influence adaptive evolutionary outcomes includes the “arrival-of-the-frequent” effect [14], which captures the simple fact that natural selection can only act on the structures that are introduced sufficiently frequently into the population through random mutations, see also [15]. Depending on the relevant time scales and mutation rates, concepts such as “free-fitness” [16, 17], or the “survival of the flattest” [18] are similarly predicted to favor the evolution of high-frequency structures.

While molecular GP maps such as the RNA model above can be interpreted as a stripped-down version of developmental bias [19, 20], historically much of the interest in the effects of bias on the arrival of variation has focused on morphological evolution. Could simplicity bias also have a dramatic impact on this larger scale? A recent study of an abstract morphological model of tissues found that random developmental mechanisms are more likely to be associated with simple morphologies and moreover, that complex morphologies are less robust to parameter changes [21]. Similarly, in a model of digital organisms [22], it was found that simple phenotypes are generated by a higher number of genotypes and are more likely to evolve from another phenotype. Higher phenotypic frequencies for simpler phenotypes were also found in a model of digital logic gates [23, 24], Boolean threshold models for gene regulatory networks [25] and a highly simplified model of neural development [26]. As a further example, models based on Lindenmeyer systems, a recursive model that can generate plant-like shapes [27] or sequences of symbols, indicate that simple phenotypes are more robust to mutations [28] and have higher neutral set sizes [5].

**Fig 2:**
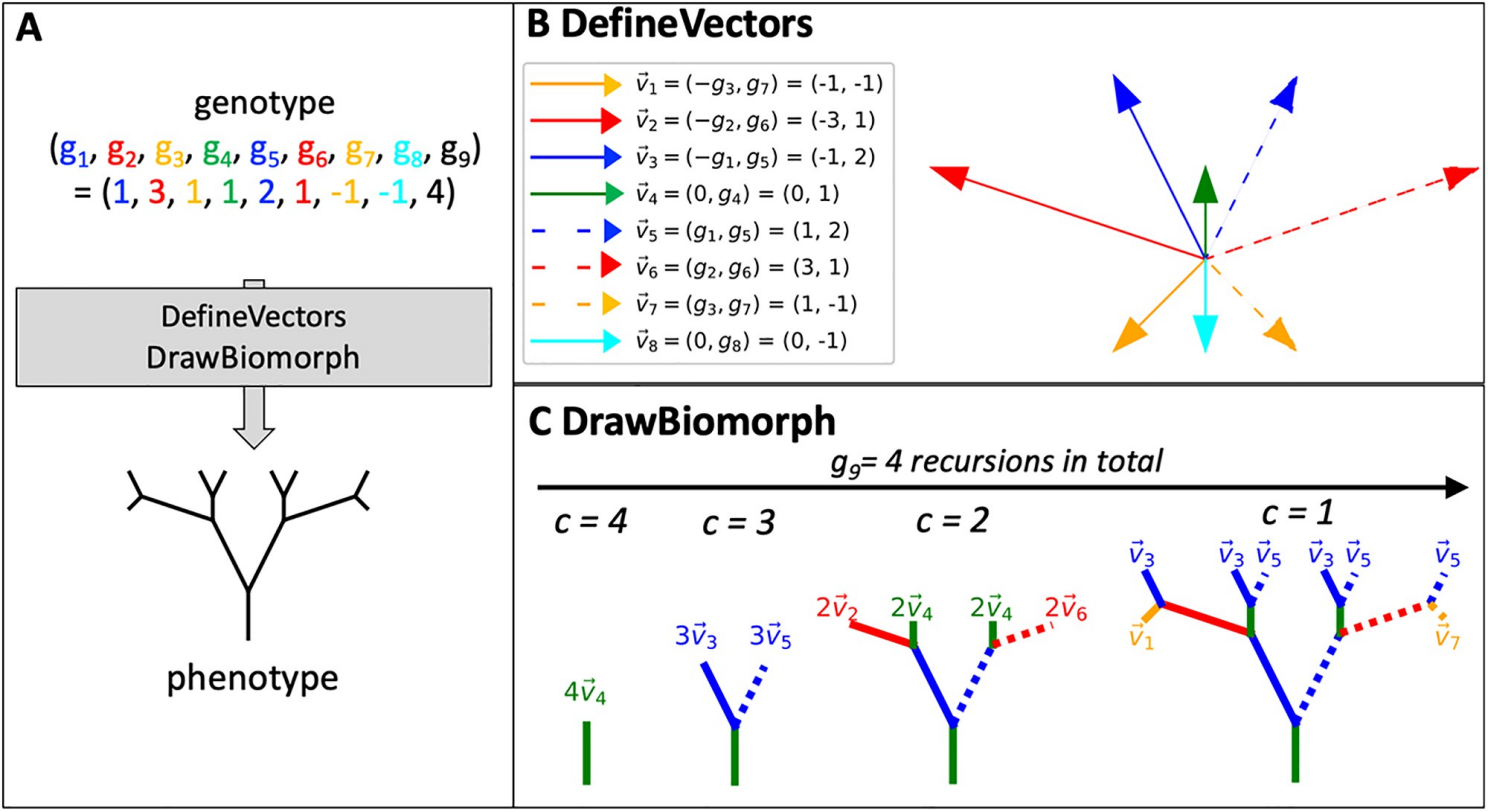
The biomorphs GP map. (A) Following [1, 29] each genotype, a set of nine integers, is used to produce the corresponding biomorph phenotype, a 2D shape using the two procedures DefineVectors and DrawBiomorph summarised in algorithm 1. (B) DefineVectors procedure: the integers at the first eight positions of the genotype (labeled *g*_1_ to *g*_8_) are used to define eight two-dimensional vectors, ${\vec{v}}_{1}$ to ${\vec{v}}_{8}$. (C) DrawBiomorph procedure: The 2D biomorph shape is created from these vectors recursively, with the number of recursions set by the integer at the ninth position of the genotype, *g*_9_. Here, we have *g*_9_ = 4 and thus four recursions, governed by the variable *c*. Since vector ${\vec{v}}_{8}$ would first be used in the fifth recursion, it is not used at all in this case.

In order to address the status of phenotype bias in systems beyond the molecular scale, we will focus on another important innovation from *The Blind Watchmaker* [1], a developmental model of two-dimensional shapes called *biomorphs*. As illustrated in Fig 2, these are made up of vectors, which are defined by (numeric) genotypes and combined into a biomorph phenotype in a recursive developmental process. This model produces a rich array of forms. In his book [1], Dawkins’s was able to gradually steer the evolution of biomorphs towards particular desired shapes in a relatively small number of generations by carefully choosing phenotypes that appear upon random mutations. In this way, he used biomorphs to illustrate the power of natural selection in a more complicated system than the simple “WEASEL” program. The main aim of this paper will be to analyze the generation of phenotypic variation more systematically in this system and test the hypothesis that this iconic model of morphological development also exhibits simplicity bias and other phenomena similar to those observed for molecular GP maps. We will also analyze the effect that these biases in the arrival of variation have on evolutionary dynamics.

**Fig 3:**
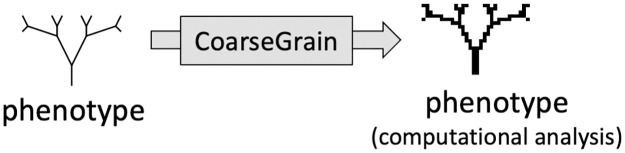
Coarse-graining biomorph figures for the computational analysis. In order to discretize the phenotypes for our computational GP map analysis, we coarse-grain the final image on a 30 × 30 grid, as illustrated for the phenotype from Fig 2.

We analyze the biomorphs GP map as follows. Firstly, to take into account the fact that many biomorph phenotypes look highly similar, we define a coarse-graining that maps them onto a discrete 30 × 30 pixel grid, as shown in Fig 3. We then exhaustively analyse all genotypes within a fixed parameter range, and use an approximate descriptional complexity measure [30] to show that the frequency-complexity relationship of biomorph phenotypes is indeed consistent with the simplicity bias of Eq 1. We show that the GP map of biomorphs exhibits many other properties that resemble those commonly found in molecular GP maps, as reviewed in [7, 31]. For example, the phenotype robustness *ρ*_*p*_, defined as the mean mutational robustness of all genotypes *g* that generate to a given phenotype *p*, scales as the logarithm of the frequency *f*_*p*_ of the phenotype. Evolvability, a measure that counts how many novel phenotypes are accessible by point mutations, correlates negatively with the mutational robustness *ρ*_*g*_ of an individual genotype *g*, but positively with phenotype robustness *ρ*_*p*_ of the whole neutral set [32]. We can rationalize these effects in the biomorphs systems as in existing GP maps, where they are captured by a simple analytically tractable model based on separating genotypes into constrained and unconstrained portions [33–36].

Another big question is to what extent these structural GP map characteristics, which determine the spectrum of novel variation that appears upon random mutations, affect evolutionary outcomes when natural selection is also at play. We first show that in the absence of selection, biases in phenotypic frequencies (which are calculated on a uniform random sampling of genotypes) are reflected in the average rates with which each biomorph phenotype appears in an evolving population. Next, we turn to a scenario that is adapted from refs [14, 15] and includes both variation and selection: Two adaptive phenotypic changes are possible and for a range of fitness values, we find that the more frequent phenotype fixes first even though it is not the fittest phenotype. We also study a scenario from Dawkins’s book [1] where he finds it hard to reconstruct an evolutionary pathway to an ‘insect’-shaped phenotype. He argues that for such rare phenotypes, while short paths exist, these are only a tiny fraction of a much larger set of potential paths, and so they are hard to reliably find. We illustrate these shortest paths and note that if neutral mutations are included, fewer phenotypic changes are needed, making it easier to create fitness functions that lead to monotonically increasing fitness paths to the final desired phenotype.

**Algorithm 1** From genotype (*g*_1_, *g*_2_, *g*_3_, *g*_4_, *g*_5_, *g*_6_, *g*_7_, *g*_8_, *g*_9_) to a biomorph drawing (paraphrased from ref [29])

**procedure** DefineVectors(*g*_1_, *g*_2_, *g*_3_, *g*_4_, *g*_5_, *g*_6_, *g*_7_, *g*_8_) ⊳ This function produces eight vectors {${\vec{v}}_{i}$} from the first eight genome positions: *g*_1_ to *g*_8_.

 ${\vec{v}}_{1}\leftarrow \left(-g_{3},g_{7}\right)$

 ${\vec{v}}_{2}\leftarrow \left(-g_{2},g_{6}\right)$

 ${\vec{v}}_{3}\leftarrow \left(-g_{1},g_{5}\right)$

 ${\vec{v}}_{4}\leftarrow \left(0,g_{4}\right)$

 ${\vec{v}}_{5}\leftarrow \left(g_{1},g_{5}\right)$

 ${\vec{v}}_{6}\leftarrow \left(g_{2},g_{6}\right)$

 ${\vec{v}}_{7}\leftarrow \left(g_{3},g_{7}\right)$

 ${\vec{v}}_{8}\leftarrow \left(0,g_{8}\right)$

**end procedure**

**procedure** DrawBiomorph(*i*, *c*, *x*_0_, *y*_0_, {${\vec{v}}_{i}$}) ⊳ Call this function with *i* = 4, *c* = *g*_9_, *x*_0_ = *y*_0_ = 0, and the vectors {${\vec{v}}_{i}$} from DefineVectors to draw the figure.

 **if** *i* = 0 **then**    ⊳ ensure that the vector index *i* is between 1 ≤ *i* ≤ 8

  *i* ← 8

 **else if** *i* = 9 **then**

  *i* ← 1

 **end if**

 (*x*_new_, *y*_new_) ← (*x*_0_, *y*_0_) + $c\times {\vec{v}}_{i}$    ⊳ add *c* times vector i to the current point

 Draw a line from (*x*_0_, *y*_0_) to (*x*_new_, *y*_new_)

 **if** *c* > 1 **then**    ⊳ recursion: start function from (*x*_new_, *y*_new_)

  DrawBiomorph(*i* − 1, *c* − 1, *x*_new_, *y*_new_)    ⊳ once with vector ${\vec{v}}_{i-1}$; *c* − 1 recursions remaining

  DrawBiomorph(*i* + 1, *c* − 1, *x*_new_, *y*_new_)    ⊳ once with vector ${\vec{v}}_{i+1}$; *c* − 1 recursions remaining

 **end if**

**end procedure**

## Materials and methods

### Dawkins’s biomorphs model

In Dawkins’s biomorphs model [1, 29], phenotypes are two-dimensional figures, recursively constructed from genotypes, which consist of nine genes *g*_1_ to *g*_9_, represented by integer values. This construction is performed in two steps (Fig 2 and Algorithm 1): first, a set of eight vectors is constructed from the genotypic information and then these vectors are combined recursively to form the final figure, as described in [29]:

1. **DefineVectors procedure** The x- and y-coordinates of eight two-dimensional vectors are set by the values of the first eight genes, *g*_1_ to *g*_8_, as shown in Fig 2B. The allocation of specific genes to vector components is fixed by Dawkins’s definition of the biomorphs system, as described in [29] (note however that we use a different indexing convention that highlights the symmetry of the figure).
2. **DrawBiomorph procedure:** The eight vectors, ${\vec{v}}_{1}$ to ${\vec{v}}_{8}$, form the basis of a recursive developmental process, where vectors are added to the figures in several stages. The ninth gene determines after how many stages this process terminates.

In order to exhaustively analyze the GP map computationally, we restrict the values in the genotypes to a finite range. We take 7 values for each of the ‘vector genes’ (−3 ≤ *g*_*i*_ ≤ 3 for *i* ∈ [1, ‥, 8]) and 8 values for the ninth gene (1 ≤ *g*_9_ ≤ 8). In this range, there are 7^8^ * 8^1^ = 46, 118, 408 genotypes. This range is somewhat smaller than the values in Dawkins’s examples [1], but they are near the limit of what is feasible for exhaustive enumerations. We chose a slightly higher range for the ninth gene than for the first eight genes since changes in the ninth gene affect the number of drawn lines and therefore have the greatest qualitative effect. The effect of extending these ranges further can be investigated with the approximate analytic model introduced in this paper. We find that the qualitative observations are unchanged (section C.3 in S1 Text).

Following Dawkins’s program of artificial evolution [1], a point mutation can increase or decrease a single gene by one integer step. This is a key difference from models like RNA, where each nucleotide can be exchanged for any other nucleotide.

### Quantifying the biomorphs GP map

We use two different approaches to study the relationship between biomorph genotypes and phenotypes on a large scale. The first approach is computational: we simply consider all genotypes within a fixed range and generate their phenotypes computationally. In order to be able to manipulate, analyze and compare the phenotypes, we coarse-grain them on a 2D grid, as explained below. The second approach is an analytic model based on separating the genome into constrained and unconstrained parts, a simplification which makes it possible to analytically calculate some key properties of a GP map [33, 34, 36].

#### Computational model with discrete phenotypes

For our computational analysis, we need a clear definition of when two biomorphs share the same phenotype. This definition should mimic the conditions in the original evolution experiments by Dawkins’s [1], who applied artificial selection based on the entire appearance of a biomorph (rather than just a specific feature). Moreover, the biomorphs were drawn on a computer screen of limited size, such that very small features may have appeared indistinguishable. Thus, biomorphs should only be treated as distinct phenotypes if they display clear visual differences. To reproduce this delineation, we project the 2D shape onto a limited-resolution 30 × 30 pixel grid as illustrated in Fig 3. In detail, this procedure works as follows:

- First, we go through the lines and merge any coinciding line segments (i.e. if the identical line segment is drawn as part of two longer lines, only one instance is kept). We only work with one half of the biomorph since the other half is given by axial symmetry.
- Secondly, we place the lines on the grid—the lines are scaled such that the total size of the grid is 5% larger than the longer dimension of the biomorph shape (either width or height) and the biomorph is placed at the center of the grid.
- Next, we record, how many lines are contained within each pixel on the grid as follows: we simply compute the total length of all line elements within the pixel (for computational reasons, we round to the nearest 10^−3^ in our calculations). Lines coinciding with the outer boundary of a pixel are assumed to contribute half their length to the pixels on either side of the boundary.
- Finally, we go through each pixel: if the total line length contained within the pixel is ≥ 20% of the side length of the pixel, the pixel value is set to one. Otherwise, it is set to zero.

This coarse-graining method has two parameters: the grid resolution (30 × 30) and the threshold for setting a pixel to one (≥20% of the length of the side of the pixel), but the qualitative characteristics of the GP map are robust to changes in these two parameters (see section C in S1 Text). To draw a coarse-grained phenotype, we simply take one genotype with this phenotype and apply algorithm 1.

To estimate the descriptional complexity $\tilde{K}\left(p\right)$ of a phenotype in this coarse-grained representation, we use the block decomposition method [30], which is designed for 2D binary arrays like our coarse-grained phenotypes. We only consider one half of the phenotype since all biomorphs are axially symmetric and use default parameters in the block decomposition method except for the choice of boundary conditions, for which we choose the sliding window approach, since the default would be to ignore pixels at the boundary in the complexity calculations.

#### Analytic model based on sequence constraints

It has been possible to analytically calculate many properties of GP maps [33, 34, 36] within an approximation that separates a genotype into constrained and unconstrained positions. The simplest versions of these approximations rely on the fact that mutations at certain positions of the genotype have no effect on the phenotype [33]. These positions are called ‘unconstrained’. Those parts of the genotypes that do affect the phenotype when they are changed are called ‘constrained’.

This technique of sequence constraints can be applied to the biomorphs as follows: The first eight sites in the biomorph genotype encode eight vectors, but not all of these vectors are used in the final shape if the developmental process terminates after a small number of stages, as dictated by gene 9 (Fig 2). Therefore, there are unused vectors and the positions of the genotype that encode such vectors must be fully unconstrained since mutations to these positions can have no effect on the phenotype. In our analytic calculations, we assume that all other positions, i.e. positions that affect one or more of the vectors in the final shape in some way, are fully constrained, i.e. that any change in these positions leads to a phenotypic change: this is a simplifying assumption since it is possible that two lines in the biomorph shape are drawn on top of one another, and in this case deleting a piece from one of these lines has no visible phenotypic effect. Thus, this analytic model is only perfectly accurate for a very detailed phenotype description: in the analytic model, any small change in any drawn line corresponds to a phenotypic change. Even if a line that was previously drawn multiple times is now only drawn once, this corresponds to a phenotypic change in the analytic model, and if the shape is rescaled, this also corresponds to a phenotypic change. Thus, the analytic description would be 100% accurate if the biomorphs are drawn with a fixed length scale on a very large screen, if lines that are generated multiple times in the developmental process are drawn as thicker lines, and if length-zero lines are included, for example as a visible dot.

Having determined which sites are constrained and which are unconstrained, we can make analytic predictions for GP map characteristics, such as phenotype frequencies, robustness, and evolvability values (see section A in S1 Text for detailed derivations). The analytic model complements the computational results since both rely on opposite assumptions: the computational model uses coarse-graining, whereas the analytic model is (overly) fine-grained. In order to compare the data from the two approaches, we restrict the genotypes to the same range of integers in both cases throughout the main text. However, since calculations in the analytic approach are fast, we also use this approach to investigate how the biomorphs GP map would change if we allowed the integer values in the genotype to vary over a wider range. This modification produces qualitatively similar outcomes, as shown in section C.3 in S1 Text.

### Models of evolving populations

To model populations of biomorphs evolving over time, we use the Wright-Fisher model with selection [38] in combination with a GP map, as done, for example, in refs. [14, 39]. The fitness of a specific genotype is calculated by mapping it to its phenotype and then using a phenotype-fitness relationship that is fixed for each simulation. We study two main scenarios. Firstly, a fitness value of one for every phenotype in the flat landscape of scenario 1, and secondly, zero fitness for every phenotype in scenario 2, except phenotypes *p*_0_, *p*_1_ and *p*_2_ which have fitness values of 1, 1 + *s*_1_ and 1 + *s*_2_ respectively, where the *s*_*i*_ are selection coefficients. Mutations occur at a constant rate *μ* per site at each generation [14]. As an initial condition, we choose a random genotype out of all genotypes that meet the specifications (for example map to a given phenotype) and initialize all individuals with this genotype. To ensure that this choice of initial conditions does not affect our measurements, we follow previous work [14] and, for a population of size *N*, let the initial population evolve for 10*N* generations before starting any measurements.

## Results

### Phenotype bias towards simple phenotypes

#### Quantifying the strength of the bias

**Fig 4:**
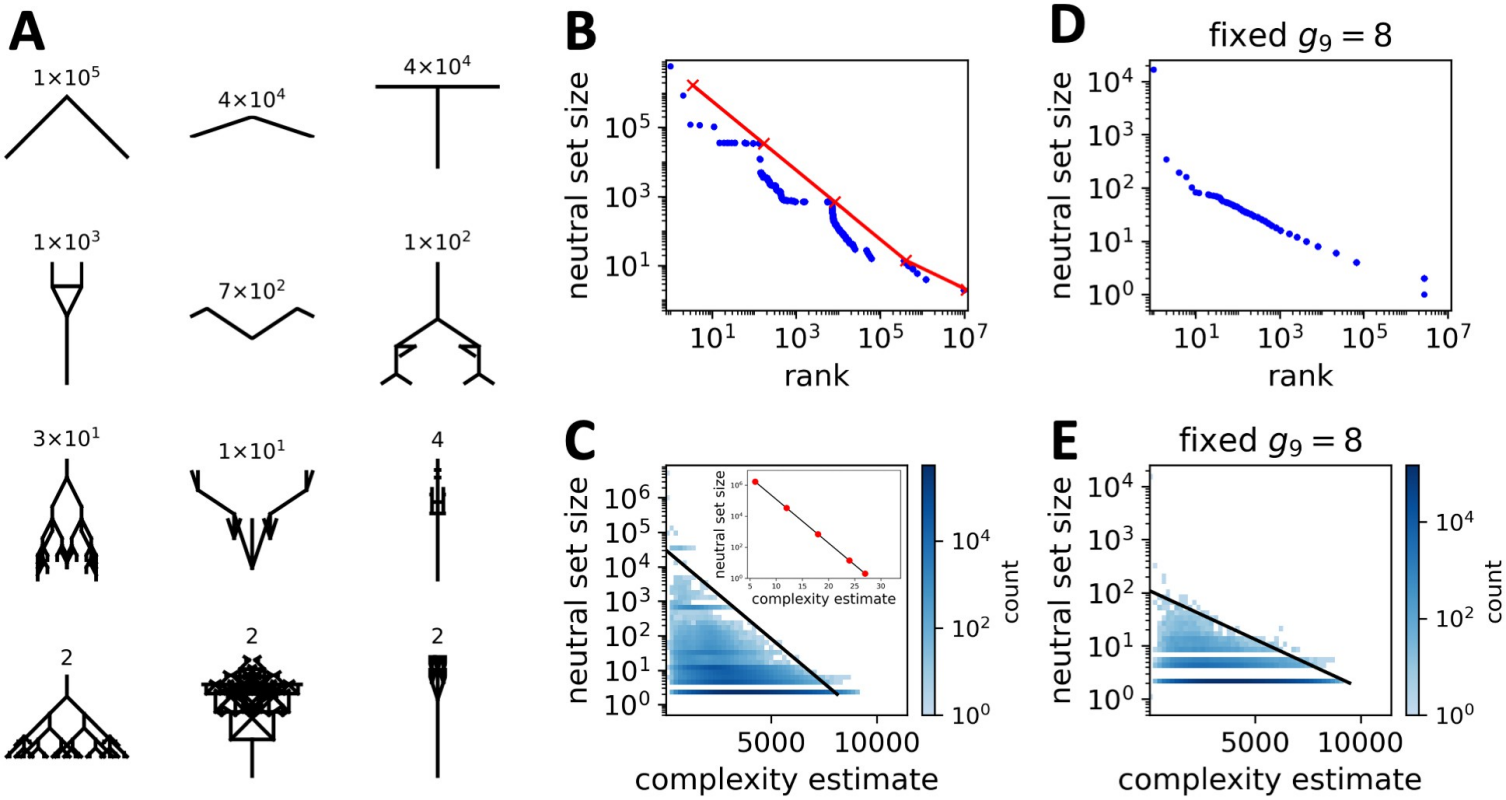
Phenotypic bias. (A) Example biomorph phenotypes: the neutral set sizes (i.e. number of genotypes per phenotype) are indicated above each image. The phenotypes shown in the first three rows are chosen to represent a range of neutral set sizes, and the last row shows three phenotypes with a neutral set size of two since ≈ 9 × 10^6^ out of ≈ 10^7^ phenotypes have this neutral set size. (B) The neutral set sizes of all phenotypes are plotted against their neutral set size ranks (i.e. the number of phenotypes with greater or equal neutral set size). The computational results are shown in blue and the analytic data of Eq 3 in red. In both treatments, neutral set sizes vary over several orders of magnitude, i.e. there is strong phenotype bias. (C) The neutral set size of each phenotype is plotted against the estimated complexity of the corresponding coarse-grained binary image (calculated using the block decomposition method [30]). The black solid line is an approximate, but not a perfect upper bound, drawn to illustrate the simplicity-bias prediction from Eq 1. Large neutral set size phenotypes tend to be low-complexity biomorphs and high-complexity biomorphs tend to have small neutral sets (deviations from the upper bound can also be understood within AIT [6]). **Inset:** Neutral set size versus complexity for the analytic model, calculated with the bound of Eq 4 which is based on an alternate complexity measure that measures the size of the constrained part of the minimal genome that generates a given biomorph. The resulting relationship is consistent with the trend in the computational results. (D) & (E) In order to test whether the results are merely a consequence of the fact that the ninth gene *g*_9_ can switch genes on or off by raising or lowering the number of stages in the growth process, we repeat the analysis with the computational method for a fixed value of *g*_9_ = 8. We find that phenotypic bias towards simple phenotypes also holds in this case.

Having introduced the relationship between biomorph genotypes and phenotypes, the first question is how many phenotypes exist and how many genotypes correspond to each of these phenotypes. In the computational results, there are ≈ 9.8 × 10^6^ different phenotypes for the 7^8^ × 8 ≈ 5 × 10^7^ genotypes that are within the parameter range considered in our analysis (approximately 1.2 × 10^7^ different phenotypes in the more fine-grained analytic model). The difference in the number of phenotypes shows that our coarse-graining is rather mild. A few examples from the computational approach are shown in Fig 4A: among these are phenotypes that are generated by approximately 10^5^ genotypes, as well as phenotypes that are only generated by two genotypes. These examples illustrate that the biomorph system exhibits strong phenotypic bias: neutral set sizes differ by several orders of magnitude between different phenotypes.

This phenotypic bias can be further observed in Fig 4B where we plot the neutral set sizes for all phenotypes. The sizes vary across more than six orders of magnitude for both the computational (blue) and the analytic (red) data. Neutral set sizes approximately follow Zipf’s law, where the relationship between neutral set size *N*_*p*_ and phenotype rank *r* (i.e. the number of phenotypes with greater or equal neutral set size) is *N*_*p*_ ∝ 1/*r* for a wide range of *N*_*p*_. This fat-tailed distribution means that most phenotypes have small neutral sets: in fact, only approximately 4 × 10^5^ out of approximately 10^7^ phenotypes have neutral set sizes greater than ten genotypes in the computational results. Note that phenotypic bias is found even without the coarse-graining introduced in the computational analysis, since it is also present in the analytic model, which does not rely on coarse-graining. From the analytic calculations (for details see section A.1 in S1 Text) we find a range of neutral set sizes that depend only on the final site of the genotype *g*_9_:

$$\begin{array}{c} N_{p}\left(g_{9}\right)\approx \{\begin{array}{cc} 2\;k^{9-2\times g_{9}}\; & \text{if}\;1\leq g_{9}\leq 4\\ 2 & \text{otherwise} \end{array} \end{array}$$ (2)

Here *k* = 7 is the number of distinct integers that are in the allowed range for genotype positions *g*_1_ to *g*_8_. Essentially the neutral set size differences in the analytic model are due to the fact that phenotypes with many unconstrained positions can be produced by a large number of genotypes [34]: each constrained site can only take one value within the entire neutral set, but each unconstrained site can take *k* different values and thus each unconstrained site leads to a larger number of distinct neutral sequences, i.e. a higher neutral set size. Specifically, each additional unconstrained site increases the neutral set size by a factor of *k*. Due to this simple relationship between constrained sites and neutral set sizes, there can only be a few phenotypes with large neutral set sizes: it is the constrained positions that define the phenotype, and since phenotypes with large neutral sets only have a small number of constrained positions, only a small number of distinct phenotypes with large neutral sets can exist. This argument gives a relationship between neutral set size *N*_*p*_ and phenotype rank *r* that closely resembles a Zipf’s law (derivation in section A.2 in S1 Text), as in some previous constrained-unconstrained models [34], and is plotted in Fig 4B:

$$\begin{array}{c} r\left(g_{9}\right)\approx \{\begin{array}{cc} k^{8}/N_{p} & \text{if}\;N_{p}>2\\ 2\times k^{8} & \text{if}\;N_{p}=2 \end{array} \end{array}$$ (3)

However, note that simplifications were made in the derivation of this equation: the full analytic expression involved a sum over *g*_9_ and we only kept the largest term in each sum. This gives us a simple expression whose *N*_*p*_-dependence is easy to analyze, but at the cost of underestimating the true rank values.

Because the neutral set size only depends on *g*_9_ in the analytic model, there are many phenotypes with identical neutral set sizes and the same rank (since we have defined the rank as the number of phenotypes with greater or equal neutral set size), and therefore there are only five distinct data points for the analytic model in Fig 4B. In the computational data, slight differences in coarse-graining imply that some phenotypes, which have exactly the same neutral set size and a single rank in the analytic model, will have neutral set sizes that differ by a few percent and thus different ranks, which leads to a ‘step-like’ relationship in Fig 4B.

#### Quantifying the bias towards low-complexity biomorphs

As can be seen visually from the examples in Fig 4A, phenotypes with higher neutral set sizes appear to be less complex. To quantify this trend, we estimate the descriptional complexity $\tilde{K}\left(p\right)$ with the block decomposition method [30], as detailed in the Material and Methods section. We find that large-neutral-set-size phenotypes have low complexity, whereas high-complexity phenotypes have small neutral sets (Fig 4C). There are phenotypes, which are simple and rare, but we do not find phenotypes that are both complex and frequent. Therefore, the GP map is biased towards a subset of simple biomorph phenotypes. This observation of an upper bound as in Eq 1, with many phenotypes below the bound, matches the AIT-based predictions by Dingle et al. [5, 6]. The biomorphs GP map, therefore, presents very similar simplicity bias phenomenology to that found for molecular GP maps in [3]. This conclusion remains unchanged when using a different, Lempel-Ziv-based complexity estimator from [5] (section D.2 in S1 Text).

In the analytic model, we cannot quantify the visual appearance of a phenotype. Instead, we approximate the complexity of a phenotype by measuring the complexity of a minimal genotype that generates the phenotype. Since not all vectors are used in the final phenotype construction, some are irrelevant and this (unconstrained) part of the genotype has no direct effect on the phenotype. Thus, the full information on the phenotype is contained within the constrained part of the genotype (if the biomorphs construction process is known), and the length of this part of the genotype $\tilde{K}$ can be used to estimate an upper bound on the description length and hence the complexity. As we have discussed, phenotypes with fewer constrained sites have exponentially higher neutral set sizes. Therefore, the analytic calculations (section A.3 in S1 Text) give the following upper bound for neutral set sizes *N*_*p*_ for a phenotype of complexity $\tilde{K}$ (again with *k* = 7 for the range of values per site):

$$\begin{array}{c} N_{p}\leq 2k\times 2^{9-\tilde{K}/3} \end{array}$$ (4)

This analytic complexity bound matches the qualitative observation of the computational data (inset of Fig 4C): complex phenotypes have small neutral set sizes, whereas simple phenotypes can have large neutral set sizes. Qualitatively, the conclusions also hold when we quantify the complexity by the number of lines in the biomorph (section D.3 in S1 Text), but the shape of the relationship differs from a simple log-linear curve in this case.

We note that most of the phenotypes Richard Dawkins’s discusses in his book [1] (for example the ones shown as illustrations) are complex phenotypes, which we estimate to have low neutral set sizes. If all phenotypes of relevance have the same neutral set sizes of (*N*_*p*_ ≈ 2), then there is no bias among these phenotypes. However, in the more general case, where there are no restrictions on which phenotypes evolve, the biases have to be taken into account.

#### Phenotype bias and simplicity bias for biomorphs with a fixed final gene 9

Our analytic calculations reveal one key reason for the phenotype bias and simplicity bias in the biomorphs GP map: phenotypes with lower values of the ninth gene have fewer developmental stages, which means that they have more unconstrained sites and thus larger neutral sets. Their lower number of developmental stages means that they contain fewer vectors and thus a lower complexity bound, thus giving a log-linear upper bound on the complexity-frequency relationship, as in Eq 1.

To test if simplicity bias is observed beyond these simple sequence-constraint effects, we restrict the value of the ninth gene to a constant: in this case, the analytic model would predict that each phenotype has the same neutral set size and same maximum complexity. However, a more detailed analysis reveals that neutral set size differences can still exist: for example a single ‘vertical line’ phenotype can be generated in many ways, by (overlapping) lines of different lengths as long as all x-components are zero, whereas other shapes will impose stricter constraints on the relative length of the different vectors. These effects are too complex to capture analytically, and we have to rely on our computational data. We find that even when *g*_9_ is held constant, and the simple sequence-constraint-based arguments no longer apply, we still observe phenotypic bias (Fig 4D for *g*_9_ = 8, Fig M in S1 Text for further values of *g*_9_) towards simple phenotypes (Fig 4E for *g*_9_ = 8, Fig N in S1 Text for further values of *g*_9_). Thus, the biomorphs GP map displays simplicity bias even in the absence of sequence-constraint-based effects.

### Further GP map structure that shapes phenotypic variation

Fundamentally, the GP map determines how random mutations produce novel variation. Many molecular GP maps have been shown to share a series of structural features beyond simplicity bias that also shape the spectrum of phenotypic variation [7, 31]. This finding prompts the question of whether the biomorph GP map also exhibits these other features.

We will focus on three structural features of GP maps that affect evolutionary dynamics. We first explore mutational robustness which quantifies the likelihood of neutral mutations that keep the phenotype unchanged. Secondly, we study how the mutational robustness of a phenotype correlates with a measure of evolvability that counts how many different unique phenotypes are accessible by point mutations. Thirdly, we analyze the phenotypic mutation probabilities, which measure how likely a mutation lead to a specific new phenotype. The definitions of these quantities follow standard practice [14, 31, 32, 40], and are given in Table 1.

**Table 1:** Definitions of key quantities for GP maps. Each line describes one quantity with a symbol and the definition. These definitions are commonly used in the literature [7, 14, 31, 32, 40, 41]. For clarity, we use tildes to distinguish genotypic quantities from corresponding phenotypic definitions.

*N*_*p*_	The neutral set size of a phenotype *p* is the number of genotypes that generate *p*.
*f*_*p*_	The phenotype frequency *f*_*p*_ of a phenotype *p* is the probability that a randomly selected genotype corresponds to the selected phenotype *p*. It is thus a normalized measure of the neutral set size *N*_*p*_ of *p*.
${\tilde{\rho }}_{g}$	The genotype robustness ${\tilde{\rho }}_{g}$ of a genotype *g* is the probability that a random mutation on *g* does not lead to a change of phenotype.
*ρ*_*p*_	The phenotype robustness *ρ*_*p*_ of a phenotype *p* is the mean genotype robustness of all genotypes *g* that correspond to phenotype *p*.
*ϕ*_*pq*_	The phenotype mutation probability *ϕ*_*pq*_ from phenotype *q* to phenotype *p* is the probability that a random mutation on a random genotype in the neutral set of *q* leads to a phenotypic change to phenotype *p*.
${\tilde{\epsilon }}_{g}$	The genotype evolvability ${\tilde{\epsilon }}_{g}$ of a genotype *g* is the total number of distinct phenotypes that can be obtained from genotype *g* through a single mutation.
*ϵ*_*p*_	The phenotype evolvability *ϵ*_*p*_ of a phenotype *p* is the total number of distinct phenotypes that can be obtained from any genotype in the neutral set of *p* through a single mutation.

To help quantify these structural features, we use a *random null model* from ref [40] where the neutral set sizes of each phenotype are kept fixed, but the individual assignments of the genotypes to phenotypes are randomized. Comparing to this random null model helps clarify where properties arise from the non-trivial structure in the GP map.

#### Phenotype robustness is high due to genetic correlations

Mutational robustness can be quantified in several ways. Firstly, genotype robustness ${\tilde{\rho }}_{g}$ describes what fraction of mutations is neutral for a given genotype *g* [32]. To characterize the robustness of a given phenotype *p*, the phenotype robustness *ρ*_*p*_ of phenotype *p* is defined by averaging the genotype robustness over the neutral set of phenotype *p* [32].

**Fig 5:**
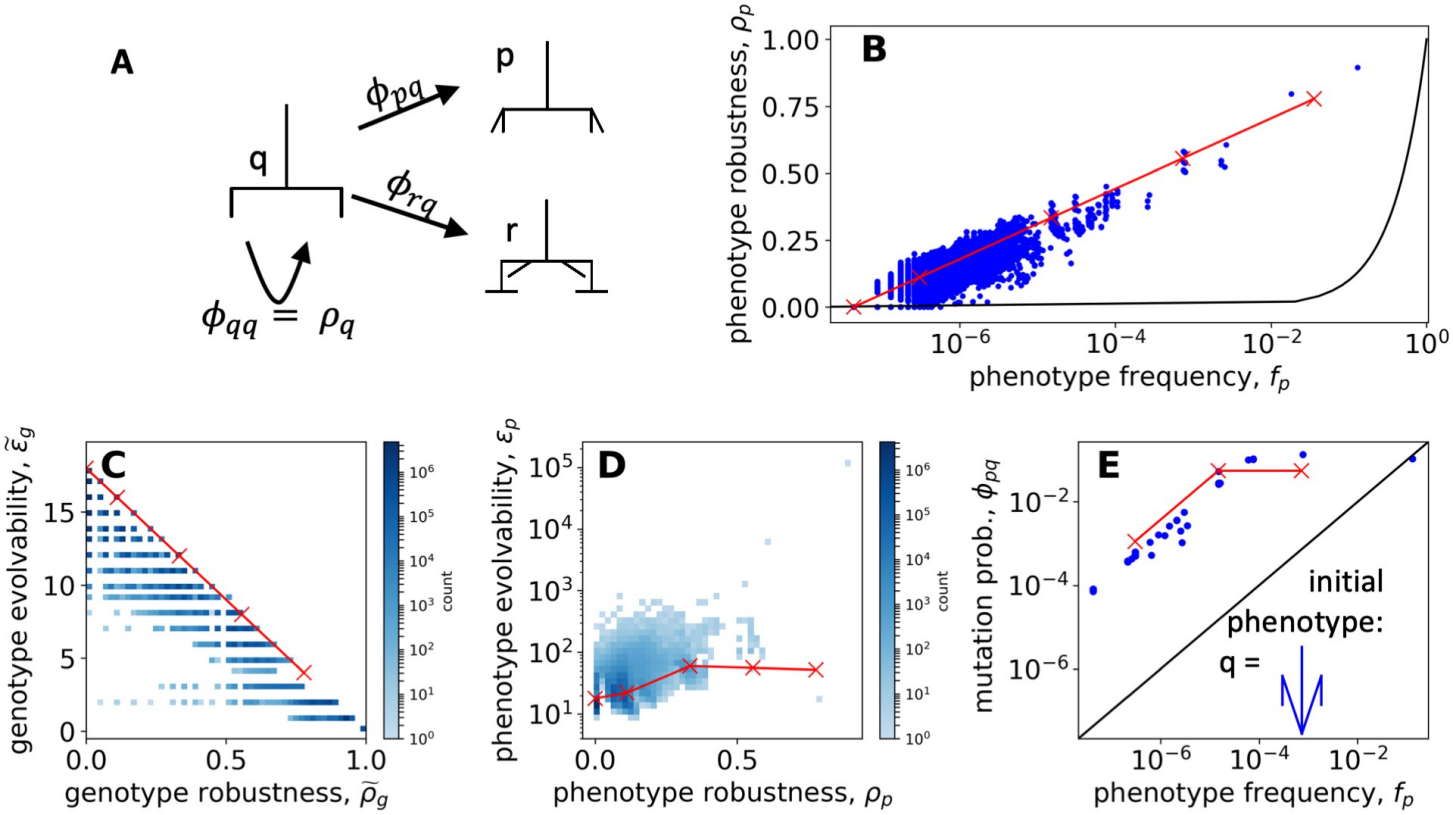
Structure in the GP map—The phenotypic effect of mutations. In every panel, the computational results are shown in blue and the analytic relationships from the constrained-unconstrained model are shown as red lines, with markers indicating the discrete allowed values. (A) Point mutations of a genotype with initial phenotype *q* can either leave *q* intact or lead to a phenotypic change to a new phenotype *p*. The likelihood of the first outcome is given (on average) by the phenotype robustness of *q*, *ρ*_*q*_; the likelihood of the latter outcome is given by the mutation probability from *q* to *p*, denoted as *ϕ*_*pq*_ (see Table 1). (B) Phenotype robustness *ρ*_*p*_ vs. phenotype frequency *f*_*p*_: the computational results (blue) are compared to the analytic calculation of Eq 5 (red). The black line (*ρ*_*p*_ = *f*_*p*_) shows the prediction from the uncorrelated null model from ref [40]. The robustness is much higher than this random null model, i.e. there are genetic correlations. (C) Genotype evolvability ${\tilde{\epsilon }}_{g}$ vs genotype robustness ${\tilde{\rho }}_{g}$ for both the computational (blue) and analytic (red, Eq 6) approach: we find the expected trade-off between robustness and evolvability at the genotype level. (D) Phenotype evolvability *ϵ*_*p*_ vs phenotype robustness *ρ*_*p*_ for both the computational (blue) and analytic (red, Eq 7) approach. As observed more widely [32], robust phenotypes have large neutral sets and are connected with many other neutral networks, so there is a positive correlation. (E) Mutation probability *ϕ*_*pq*_ vs. phenotype frequency *f*_*p*_ for a fixed initial phenotype q (shown in the corner). Again, the computational data is shown in blue, the analytic data in red (given by a parametric equation from section A.7 in S1 Text), and the black line shows the null model from ref [40], which gives *ϕ*_*pq*_ = *f*_*p*_, Data points with *ϕ*_*pq*_ = 0 are excluded due to the logarithmic scale, even though 99.997% of all biomorph phenotypes have *ϕ*_*pq*_ = 0 for this particular initial phenotype *q*.

In the simple null model with a random assignment of phenotypes to genotypes, one would expect that a mutation on a genotype *g* with phenotype *p* would generate the same phenotype with a probability proportional to the phenotype frequency *f*_*p*_ of *p* [40]. This null expectation is plotted by a solid black line in Fig 5B. However, as can be seen in the same figure, we find a completely different scaling, namely that *ρ*_*p*_ ∝ log(*f*_*p*_) ≫ *f*_*p*_. This is seen both in the computational results (blue) and in the analytic (red) calculations. In the analytic calculations, we can rationalize this as follows: each unconstrained site contributes a constant amount of robustness since it can vary freely without changing the phenotype. However, it contributes multiplicatively to the neutral set size since the values at unconstrained sites can be combined in different ways to generate genotypes within the neutral set. Taken together, this gives a log-linear relationship, which is derived in section SA.4 in S1 Text:

$$\begin{array}{c} \rho_{p}\approx 1/9\times {\text{log}}_{k}\left(k^{8}\times 8\times f_{p}/2\right) \end{array}$$ (5)

Note that robustness values in the analytic model are discrete because neutral set sizes and hence phenotype frequencies are discrete in Eq 2: the allowed values are *ρ*_*p*_ = 0 and *ρ*_*p*_ = (1 + 2*n*)/9 with integer *n* in the range 0 ≤ *n* ≤ 3.

This log-linear scaling of the robustness and frequency has been reported in many other GP maps [7], including the RNA secondary structure GP map [40, 42, 43], Boolean threshold models for gene regulatory networks [25], a multi-level GP map model called Toylife [44], the polyomino GP map for protein quaternary structure [40, 45], the HP model for protein tertiary structure [40], empirical data on sequences binding transcription factors and RNA binding proteins [46] and is typical of sequence-constraint-based models [33], a class of models that includes our analytic model for the biomorphs system. It may hold for a wider set of input-output maps as well [47], and is close to the maximum possible robustness for this class of systems [48]. Because robustness is higher than in the null model in all of these cases, two genotypes that differ only by a single mutation are more likely to correspond to the same phenotype than two randomly chosen genotypes. Such deviations from the (correlation-free) null model have been referred to as *genetic correlations* [40]. The high robustness provided by genetic correlations means that evolving populations can much more easily explore a neutral network than in an uncorrelated model [40], implying enhanced navigability of fitness landscapes [49].

#### Genotype robustness and evolvability are negatively correlated

We next analyze the link between mutational robustness and non-neutral mutations. It is clear that there must be a trade-off on the genotypic level [32]. There are only a fixed number of possible mutations per genotype and the more that are neutral, the fewer non-neutral mutations are possible. This trade-off can be quantified by defining the genotype evolvability ${\tilde{\epsilon }}_{g}$ as the total number of distinct phenotypic changes that are possible through random mutations starting from a given genotype [32]. In Fig 5C we illustrate this predicted trade-off between genotype robustness *ρ*_*g*_ and evolvability ${\tilde{\epsilon }}_{g}$ in the biomorphs system. This pattern is seen both in the computational results (blue) and in the analytic predictions (red) where every non-neutral mutation from a given genotype gives a distinct phenotype, leading to a simple trade-off derived in section SA.5 in S1 Text:

$$\begin{array}{c} {\tilde{\epsilon }}_{g}=18\times \left(1-\rho_{g}\right) \end{array}$$ (6)

#### Phenotype robustness and evolvability are positively correlated

In his “Robustness and evolvability: a paradox resolved” paper, Wagner [32] argued that the relationship between robustness and evolvability looks markedly different if we consider the neutral set mapping to a phenotype instead of individual genotypes. A phenotype with high robustness *ρ*_*p*_ is likely to have a large neutral set size. Even if, due to the high robustness, only a relatively small number of non-neutral mutations is possible from each of the genotypes in this neutral set, the higher the number of genotypes, the higher the number of novel phenotypic changes accessible through mutations [32]. This concept is quantified by the phenotype evolvability *e*_*p*_ of phenotype *p* (Table 1), which counts the total number of alternative phenotypes accessible from the entire neutral set. We find that, just as for other GP maps [32, 45], this argument holds for the biomorphs GP map: phenotypes with higher phenotype robustness tend to have higher phenotype evolvability. Again, this is seen both in the computational results (blue) and the analytic calculations (red) in Fig 5D.

In the analytic calculation, the positive relationship between evolvability and robustness on the phenotypic level has the following origin: genotypic changes at the unconstrained positions of *p* are neutral and thus occur within the neutral set of *p*. These changes can accumulate and contribute to evolvability because they can become important if a mutation raises the value of *g*_9_ and a new phenotype with a higher number of developmental stages emerges, for which these positions can be important. Thus, different genotypes within the neutral set of *p* can give rise to different phenotypic changes and the evolvability of the neutral set can be higher than the evolvability of an individual genotype in the neutral set. The phenotype evolvability in the biomorphs system can be higher than the genotype evolvability because unconstrained sites can become constrained (and thus phenotypically relevant) after mutations, as has been shown [36] for other abstract GP map models, including an RNA-inspired model. The full calculation in section A.6 in S1 Text gives the following relationship:

$$\begin{array}{c} \epsilon_{p}=\{\begin{array}{cc} 18 & \text{if}\;\rho_{p}=0\\ 15+k & \text{if}\;\rho_{p}=1/9\\ 18\times \left(1-\rho_{p}\right)-1+k^{2} & \text{if}\;2/9\leq \rho_{p} \end{array} \end{array}$$ (7)

While the trend is the same in the computational results (blue) and the analytic predictions (red), clear deviations between the two approaches exist in the phenotype evolvability calculation. This deviation may be partly due to the nature of the definition of evolvability: all possible phenotypic transitions *p* to *q* contribute equally to *ϵ*_*p*_, even if they are only possible from a single genotype in the neutral set of *p*. Thus, phenotype evolvability is much more sensitive to small changes in the GP map than quantities like phenotype robustness, which are given by the average over a neutral set. For example, one reason behind higher evolvability values in the computational results may be the following: the simplifying assumptions in the analytic model mean that each genotype in a given phenotype’s neutral set has the same value of *g*_9_. In the computational data however, there are counterexamples; for example any genotype with a zero for all positions that affect x-components is a vertical line, regardless of the value of *g*_9_. These additional genotypes in the neutral set of the ‘vertical line’ phenotype in the computational data could facilitate a range of additional phenotypic changes and thus lead to higher evolvability.

Note that in both the analytic calculations and the computational results, the phenotype evolvability is typically several orders of magnitude lower than the number of phenotypes (≈ 10^7^). Thus, while the number of possible phenotypic changes from the neutral set of an initial phenotype can be much larger than the number of possible phenotypic changes from a single genotype, not all phenotypic changes can be achieved in a single mutation. The reasons can be understood from a simple example: if the initial biomorph *q* contains a line pointing in the positive y-direction, at least two point mutations are needed to change this to a vector pointing in the negative y-direction (1 → 0 → −1 in the relevant gene).

We hasten to point out that the word evolvability encompasses a much broader set of concepts than the particular measure we discuss above. Evolvability [50–52] is often defined as the potential for “viable and heritable phenotypic variation” [51]. Because many different aspects of biology touch on this capacity, evolvability can be measured in many different ways [53] and thus the genotype and phenotype evolvability measures used here are just one of the ways this concept can be unpacked for biomorphs. Interestingly, although the word appears in the literature at least as far back as 1931 [54], Richard Dawkins’s famous paper on the evolution of evolvability [29], which builds on the biomorphs model, kicked off the modern use of the word [55]. In Dawkins’s paper, he notes that evolvability depends on the developmental process. He contrasts the classic biomorphs studied here with variations to the model that have additional developmental steps, such as segmentation. This perspective on evolvability differs from the one we have analyzed here, where we compare the phenotype evolvability of biomorph phenotypes that all originated from the same fixed developmental system. The rich concept of evolvability thus has many facets [53].

#### The mean probability *ϕ*_*pq*_ of a non-neutral mutation from phenotype *q* to phenotype *p* is higher for target phenotypes of high *f*_*p*_

Our phenotype evolvability calculations only tell us how many different phenotypic changes are possible, but not how likely they are. This latter concept is quantified by the phenotypic mutation probability *ϕ*_*pq*_, which measures how likely a mutation is to produce phenotype *p*, given that the phenotype before the mutation is *q* [14]. It is an average quantity computed over the neutral set of all genotypes mapping to *q*. The random null model predicts that *ϕ*_*pq*_ = *f*_*p*_, indicating that the probability of phenotype *q* mutating to phenotype *p* is largely independent of the source phenotype *q* [14, 40]. Indeed, in several molecular GP maps, such a correlation between *ϕ*_*pq*_ and *f*_*p*_ has been found, especially in cases with a high-frequency initial phenotype [40], but only as a first approximation [56].

Fig 5E plots the mutation probabilities *ϕ*_*pq*_ for an initial phenotype *q* of intermediate neutral set size (*N*_*p*_ = 3.5 × 10^3^ in the computational results, *N*_*p*_ = 6.9 × 10^2^ in the analytic model). While it is clear that for accessible phenotypes, *ϕ*_*pq*_ indeed increases with the frequency of the target phenotype *p*, the data deviates from the simple relationship *ϕ*_*pq*_ = *f*_*p*_. One deviation is that most phenotypic transitions are impossible (i.e. *ϕ*_*pq*_ = 0 and thus these *ϕ*_*pq*_ values do not appear in this log-log plot): for the initial phenotype *q* shown in Fig 5E, we have *ϕ*_*pq*_ = 0 for ≈99.997% of all possible biomorph phenotypes *p*, and this figure is even higher for other less evolvable choices of *q*—the phenotype *q* in Fig 5E, which is based on a genotype drawn at random from all genotypes with *g*_9_ = 3, has a comparatively high evolvability of 261 phenotypes in the computational results (60 in the analytic model) and thus a higher number of possible phenotypic changes than most other phenotypes. As noted in our discussion of phenotype evolvability, the fact that many phenotypic changes are impossible through single mutations is a feature of the biomorphs system, and it may not be shared across all GP maps. Interestingly, the allowed phenotypic transitions, i.e. those with non-zero *ϕ*_*pq*_, are mostly transitions to phenotypes whose phenotypic frequency is within two orders of magnitude of the phenotypic frequency of *q*. In the analytic model, this is easy to explain: each gene, including *g*_9_ can only vary by ±1 in a single mutation and thus the neutral set size, which depends on *g*_9_ (Eq 2), can only vary by a limited amount.

If we consider the possible phenotypic transitions shown in Fig 5E, we find that transitions to target phenotypes with high phenotypic frequency tend to be more likely, i.e. a higher *f*_*p*_ tends to be associated with a higher *ϕ*_*pq*_. There is a linear regime (*ϕ*_*pq*_ ∝ *f*_*p*_), but also a regime at a higher frequency where the relationship plateaus. This pattern is observed both in the computational results (blue scatter points in Fig 5E) and the analytic calculation (red line—this is given by a parametric equation derived in section A.7 in S1 Text). This parametric equation summarizes the following relationships: high *ϕ*_*pq*_ values are predicted for phenotypic changes to phenotypes with the same or fewer constrained values, which are known to have equal or larger phenotypic frequencies than the initial phenotype *q*. Low *ϕ*_*pq*_ values are predicted for phenotypic changes to phenotypes with a higher number of constrained values. These transitions are rare because they can only happen on a specific genetic background because of the additional constrained values. Since these phenotypic transitions correspond to a higher number of constrained sites, they have lower phenotypic frequencies than the initial phenotype *q*. While the computational and analytic data show good agreement, the computational data includes additional transitions at very high and very low values of *f*_*q*_: the transition with *f*_*q*_ > 10^−2^ corresponds to the simple ‘line’-shaped phenotype. This phenotype’s neutral set is highly affected by the treatment of overlapping vertical lines along the y-axis and by rescaling, and therefore shows large deviations between the two models. Similarly, the computational data contains additional transitions with low values of *ϕ*_*pq*_ and *f*_*q*_. As we argued when comparing evolvability predictions, this is because phenotypic transitions that are only possible from one or a small number of specific genotypes in the initial neutral set are particularly sensitive to a change in GP map definition. These differences between the computational and the analytic data mean that the bias in the mutation probabilities *ϕ*_*pq*_ is higher in the computational data.

Overall, our main takeaway is that most phenotypic transitions are not possible through single mutations, but out of the possible phenotypic transitions, those to phenotypes with high neutral set sizes tend to be more likely. The second aspect is in agreement with results from a series of other GP maps [40], even though the exact shape of the relationship with its two distinct regimes is different. Because complex phenotypes have low phenotypic frequencies, this implies that the more likely phenotypic changes tend to be towards lower-complexity phenotypes (as confirmed in Fig L in S1 Text). This agrees with previous research that has argued that transitions from high-complexity phenotypes to low-complexity phenotypes are more likely than the reverse, both for an L-system-based GP map [57] and a GP map for digital organisms [22]. However, it is important to note that in the biomorphs GP map these arguments only hold for initial phenotypes with a relatively high neutral set size: if the initial phenotype is one of the ≈ 9 × 10^6^ phenotypes with a neutral set size of *N*_*p*_ = 2, then there are only up to 36 possible distinct mutations (18 per genotype for *N*_*p*_ = 2 genotypes), and since typically at least ten phenotypic transitions are found among these 36 distinct mutations, all non-zero *ϕ*_*pq*_ values are of a similar order of magnitude and strong bias is impossible.

#### GP map structure for biomorphs with a fixed final gene 9

Many of the results we find here for the biomorphs model are generically found in analyses that approximate a GP map with a constrained/unconstrained sequence model [33, 34, 36]. For example a log-linear relationship between phenotypic robustness and frequency [33] and a positive correlation between phenotype robustness and evolvability [36] are easy to qualitatively understand within this picture.

In the biomorphs model, gene 9 has a special character in generating the constrained-unconstrained model. So it is natural to ask whether the use of this gene is the only reason we observe these generic behaviours. To examine this question, we study a set of models with gene 9 fixed to values ranging from 2 to 8. In our analytic model, this results in each phenotype having the same number of constrained sites, the same frequency, the same robustness and the same evolvability. However, as can be seen in section E in S1 Text, for a fixed gene 9 we still find a log-linear relationship between phenotypic frequency and robustness, a tradeoff between genotypic evolvability and robustness, a positive correlation between phenotypic evolvability and robustness and differences in mutation probabilities, such that mutations to higher-frequency phenotypes tend to be more likely. The only exceptions are cases with *g*_9_ ≤ 3, when very few phenotypes exist and analyses on the phenotypic level are not meaningful. In other words, even for a fixed gene 9, we observe the generic behaviour seen in other molecular GP maps, albeit on a smaller scale.

### Phenotype bias and adaptive evolution

Having analyzed what the GP map can tell us about the phenotypic effect of mutations in general, we next investigate how this structure in the arrival of variation affects an evolving population. Modeling evolving populations requires us to make assumptions about the way in which fitness depends on the biomorph phenotype and so we study several scenarios. All data in the following sections rely on computer simulations that use the computational treatment of the biomorphs GP map.

#### Scenario 1: Neutral evolution on a flat fitness landscape

**Fig 6:**
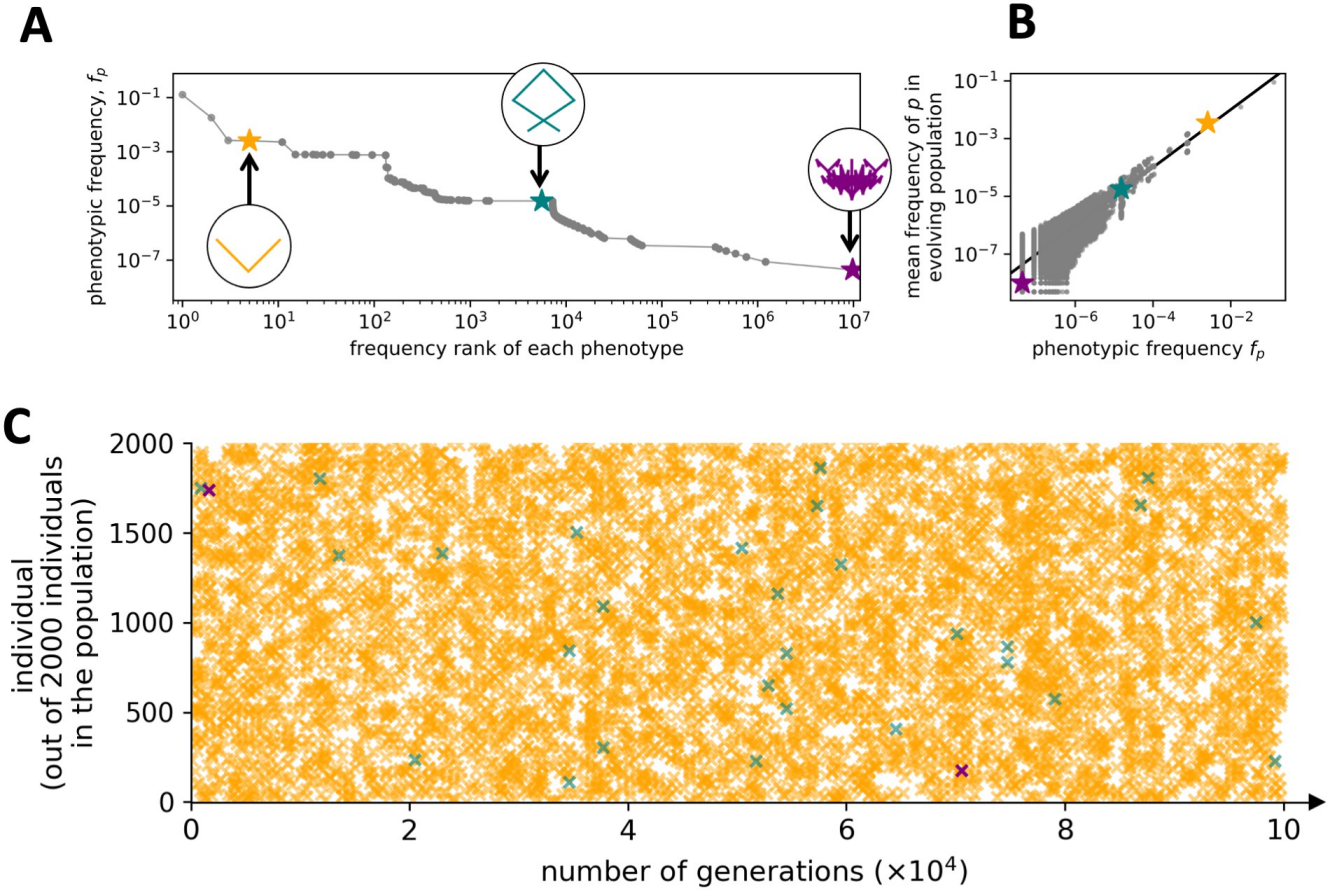
Evolution in a flat fitness landscape. We simulate a scenario where all biomorph phenotypes are equally fit so that there is no selection, just neutral drift. (A) This plot of phenotypic frequency versus rank highlights three phenotypes that are chosen for more detailed analysis: one with high frequency in genotype space (yellow), one with medium frequency (teal), and one with low frequency (purple). (B) The normalized number of times each phenotype occurs in the population is plotted against its phenotypic frequency (for all phenotypes; the chosen phenotypes are highlighted in color). As might be expected in the absence of selection, we see an approximate one-to-one correspondence (as indicated by the black line), with some fluctuations at low values of *f*_*p*_. Due to the logarithmic scale, only non-zero values are shown. (C) We plot the occurrence in the population of each of the three phenotypes highlighted in (A) once every 1000 generations. This representation highlights the relative frequencies with which the different phenotypes appear in an evolving population of 2000 individuals. The most frequent phenotype (yellow) appears in the population with an average of about 7 individuals per generation. The intermediate frequency phenotype (teal) appears in the population on average only once every 28 generations, so about 200 times less frequently than the yellow phenotype. The rarest phenotype (purple) only appears twice in all 10^5^ generations. To take this into account, we plot both times it appears, even when this is not a generation that is plotted for the other phenotypes (where we only show one every 1000 generations due to space constraints). **Parameters**: Population size *N* = 2000 individuals, with a mutation rate of *μ* = 0.1 per site, evolving for 10^5^ generations, initialized on a random initial genotype—we run the simulation for 10*N* = 20000 generations before starting the analysis to minimize artifacts of the initial conditions.

We start with the simplest scenario: a population of size *N* = 2000 evolves under Wright-Fisher dynamics without the effect of selection, i.e. all phenotypes are equally fit and there is only genetic drift. Each individual genotype in each generation could carry any of the approximately 10^7^ different phenotypes, so we simplify our analysis by focusing on three phenotypes with different neutral set sizes, as highlighted in Fig 6A. We recorded each time that one of these phenotypes was found in the population (Fig 6C). Out of these three phenotypes, the one with the highest neutral set size appears most frequently in the population, followed by the phenotype with an intermediate neutral set size. The phenotype with the lowest neutral set size only appears twice. The takeaway from this scenario is the intuitive result that, on average, the rate at which individual phenotypes appear in a neutrally evolving population is well predicted by their global phenotypic frequencies *f*_*p*_ (Fig 6B), as previously seen for molecular GP maps [3, 37]. It is not hard to imagine that these large differences in the rates can also affect adaptive evolutionary scenarios where fitness plays a role, as we will see later in this paper.

A slightly more complex version of this scenario is analysed in Fig U in S1 Text: here all tree-like biomorph phenotypes are equally fit, but all biomorphs that are not tree-like are unviable. This scenario approximates a situation where some phenotypic features are under extremely strong selection, whereas others are irrelevant for survival and therefore neutral. Qualitatively, we observe the same trends: there is phenotypic bias over several orders of magnitude among the viable phenotypes and this bias is reflected in the evolving population.

#### Scenario 2: Two peak fitness landscape

**Fig 7:**
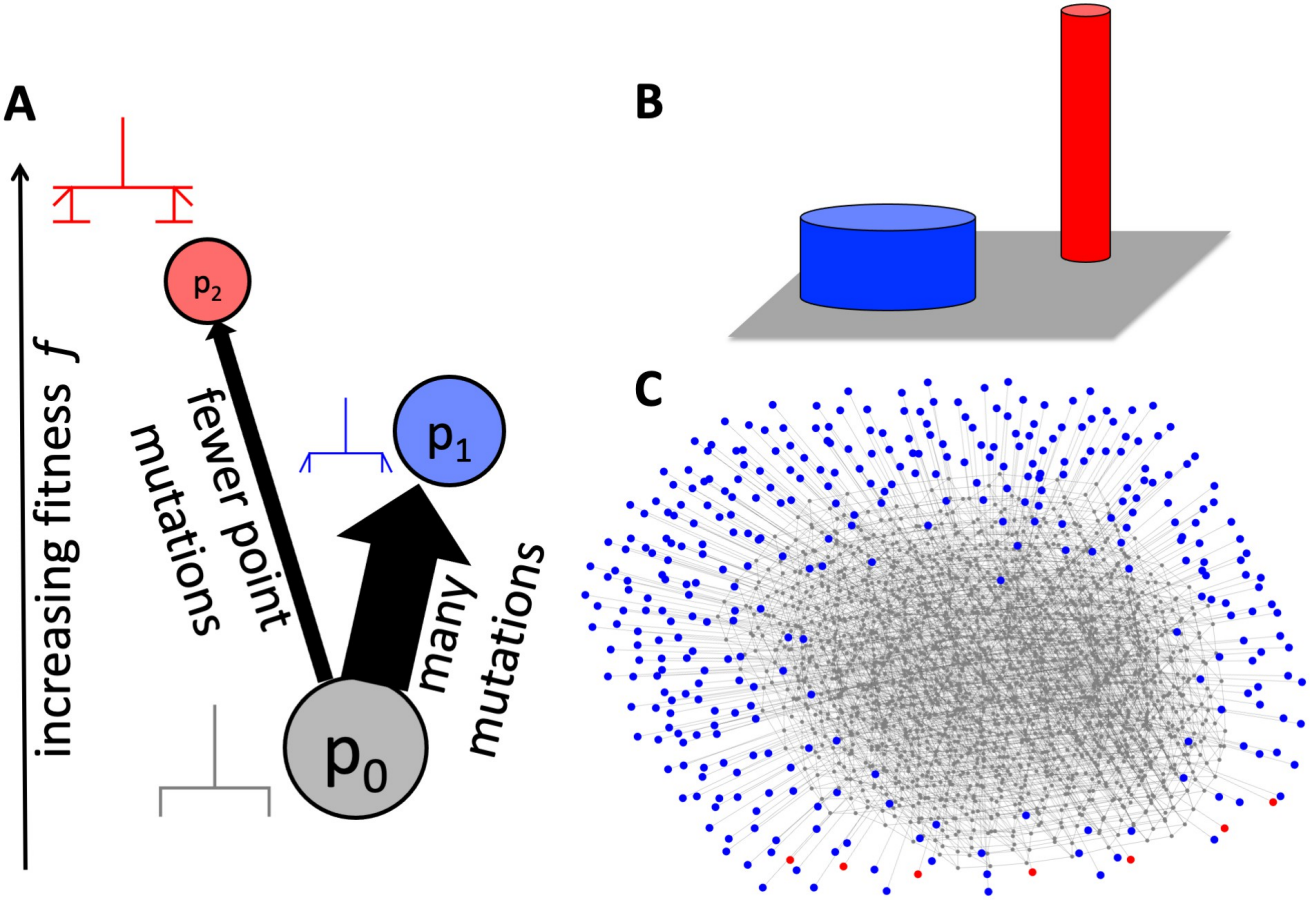
Schematic of the two-peaked fitness landscape (following a similar example for RNA [14]). (A) Only three phenotypes have nonzero fitness—the initial phenotype *p*_0_ (grey) and the two adaptive phenotypes, *p*_1_ (blue) and *p*_2_ (red). Phenotype *p*_1_ is more frequent and has a higher mutation probability $\phi_{p_{1}p_{0}}$ from *p*_0_ than *p*_2_ does, but *p*_2_ has higher fitness. A single point mutation can convert *p*_0_ into *p*_1_ or *p*_2_, but not *p*_1_ into *p*_2_. (B) The same scenario is sketched as a schematic ‘fitness landscape’—the population starts with the *p*_0_ phenotype (grey area) and there are two fitness peaks, corresponding to *p*_1_ (blue) and *p*_2_ (red). While *p*_1_ is a broader fitness peak and thus a larger mutational target, *p*_2_ is a higher fitness peak. (C) A schematic representation of the relevant part of the GP map: each genotype is drawn as a node in the color of the corresponding phenotype and two genotypes are connected by an edge if one can be reached from the other through a single point mutation. This representation illustrates that there are many different genotypes for each phenotype and that the population will therefore evolve neutrally on the neutral component of *p*_0_, until moving to either *p*_1_ or *p*_2_. Genotypes are only included in this network if they belong to the initial neutral component of *p*_0_, or if they are direct mutational neighbors of that neutral component and map to *p*_1_ or *p*_2_. The initial neutral component can be found by starting from the genotype (-2, 0, 2, -2, 0, 0, -2, -2, 3).

Next, following [14, 15], we investigate a more complex adaptive scenario, a two-peak fitness landscape, where two phenotypes have different selective advantages over an initial source phenotype. As illustrated in Fig 7, the population starts at an initial phenotype *p*_0_ and most alternative phenotypes are unviable, with two exceptions, phenotypes *p*_1_ and *p*_2_. For simplicity, the population is chosen such that we are approximately in the strong-selection weak-mutation regime, where adaptive mutations are a limiting factor. The criterion for the strong-selection weak-mutation regime is that the product of mutation rate and population size is small and the product of the population size and selective advantages large [58]: here these quantities are 9 × *μN* = 0.45 (where the factor of nine accounts for the fact that the mutation rate is per-site) and *N* × *s* ≥ 10.

In this particular example, phenotype *p*_1_ has a frequency *f*_1_ = 1.5 × 10^−5^, and phenotype *p*_2_ has a frequency *f*_2_ = 9.1 × 10^−7^ ≈ 0.06*f*_1_. However, since the initial condition is known, the relevant quantities are the probabilities of obtaining *p*_1_ and *p*_2_ through mutations from our initial conditions: the whole population is initially undergoing neutral exploration starting on one particular genotype *g*_0_ in the neutral set of *p*_0_ and can therefore drift through the entire part of the neutral set of *p*_0_ that is accessible from *g*_0_ through neutral mutations. This part is known as a neutral component (NC) of *p*_0_ [59]. The phenotype mutation probabilities for that NC determine the rates at which the two adaptive phenotypes are expected to appear [14]: these are also biased towards *p*_1_, with $\phi_{p_{1}p_{0}}\approx 9.7\times 10^{-3}$ and $\phi_{p_{2}p_{0}}=1.9\times 10^{-4}\approx 0.02\phi_{p_{1}p_{0}}$. The fitness is traditionally expressed as *F*_*p*_ = 1 + *s*_*p*_ in terms of the selection coefficient *s*_*p*_. For the neutral phenotype, *s*_0_ = 0, and we vary the two other fitnesses, but we are only interested in the non-trivial case, where the rarer phenotype has larger fitness, in other words, *s*_2_ > *s*_1_ > 0.

**Fig 8:**
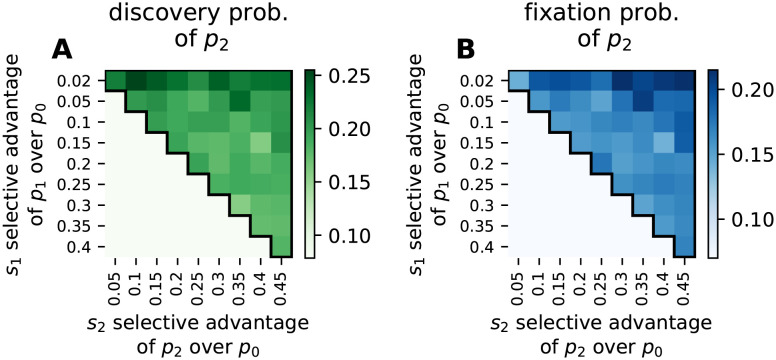
Probability of discovery and fixation of the fitter and less frequent phenotype p_2_. (A) Probability that the rarer phenotype *p*_2_ appears in the population before the first fixation event. As long as the more frequent phenotype *p*_1_ has a sufficiently high selective advantage *s*_1_ over the initial phenotype, it is likely to appear and fix quickly so that it becomes unlikely that the rarer phenotype *p*_2_ appears even once before *p*_1_ fixes. (B) The probability that the rarer phenotype *p*_2_ is the first to fix. *p*_2_ can only fix once it appears and so its fixation probability is even lower than the probabilities in (A). These results for the phenotypic bias are consistent with the trends expected from mutational bias [15]. Parameters: population size *N* = 500, mutation rate *μ* = 0.0001, 10^3^ repetitions per parameter set; we consider a phenotype to have fixed if > 70% of the individuals carry that phenotype. The populations start from randomly chosen initial genotypes *g*_0_ that all belong to the NC shown in Fig 7 and then evolve neutrally for 10*N* generations before the three-peak simulations begin.

In our simulations of the fixation dynamics, both *p*_1_ and *p*_2_ can evolve from the initial phenotype *p*_0_ and both are fitter than *p*_0_. If selection alone was the deciding factor, we would expect *p*_2_ to evolve in every simulation since it has the highest selective advantage. However, the more frequent phenotype *p*_1_ also has a selective advantage over the initial phenotype *p*_0_, albeit a smaller one, and so *p*_1_ can reach fixation before *p*_2_ appears in the population as potential variation. Since it is not possible to go from *p*_1_ to *p*_2_ through a single point mutation, but only via a two-step process from *p*_1_ back to *p*_0_ and then to *p*_2_, we focus only on the first fixation event. This is a good approximation since the population is unlikely to go back to *p*_0_ via drift due to the strong selection, as shown in section G in S1 Text. In Fig 8 we analyze how likely it is that (A) the fitter and rarer phenotype *p*_2_ has appeared at least once before the first fixation event and (B) the first fixation event is a fixation of *p*_2_.

Fig 8A shows a heatmap of the probability that the rarer phenotype *p*_2_ appears at all in the population before the first fixation event. This probability is low in the entire range of selective advantages we consider, but it increases slightly if the high-frequency, lower fitness phenotype, *p*_1_ has a low selective advantage (i.e. *s*_1_ = 0.02). This effect occurs because if the high-frequency phenotype *p*_1_ takes longer to go to fixation, this leaves more time for *p*_2_ to appear. Note that the selective advantage of the low-frequency phenotype *p*_2_ does not play a role here: *p*_2_ could be infinitely fit, but when it appears in the population for the first time is unaffected by its fitness.

It is clear that *p*_2_ can only achieve fixation if it appears in the simulation at some point, but even if it appears, it could still be lost due to stochasticity. Thus, we now turn to the probability that *p*_2_ is the first to reach fixation (Fig 8B). This probability is of a similar order of magnitude to the probability that *p*_2_ appears, indicating that *p*_2_ is likely to fix once it appears. However, since the fixation probability cannot exceed the probability of discovery, it remains low for the entire range of selective advantages we consider. Interestingly, the impact of varying *s*_1_ and *s*_2_ is not as strong here as in the original paper by Yampolsky and Stoltzfus [15] that first studied such effects, because their calculations focus on a simpler case with only three genotypes. For evolution on GP maps, where there are many genotypes mapping to *p*_0_, the constant-rate assumptions underlying existing work are merely an approximation to the true dynamics [14, 60].

To sum up, in this particular example, the higher rate with which *p*_1_ is introduced into the population due to random mutations dominates over the difference in selective advantage, which would favor *p*_2_. This does not mean that selection does not play a role: selection is the reason why each simulation leads to one adaptive fixation (*p*_1_ or *p*_2_) and due to selection the probability of a *p*_2_ fixation is highest if the selective advantage of *p*_2_ is much higher than that of *p*_1_.

#### Scenario 3: Finding Dawkins’s beetle

**Fig 9:**
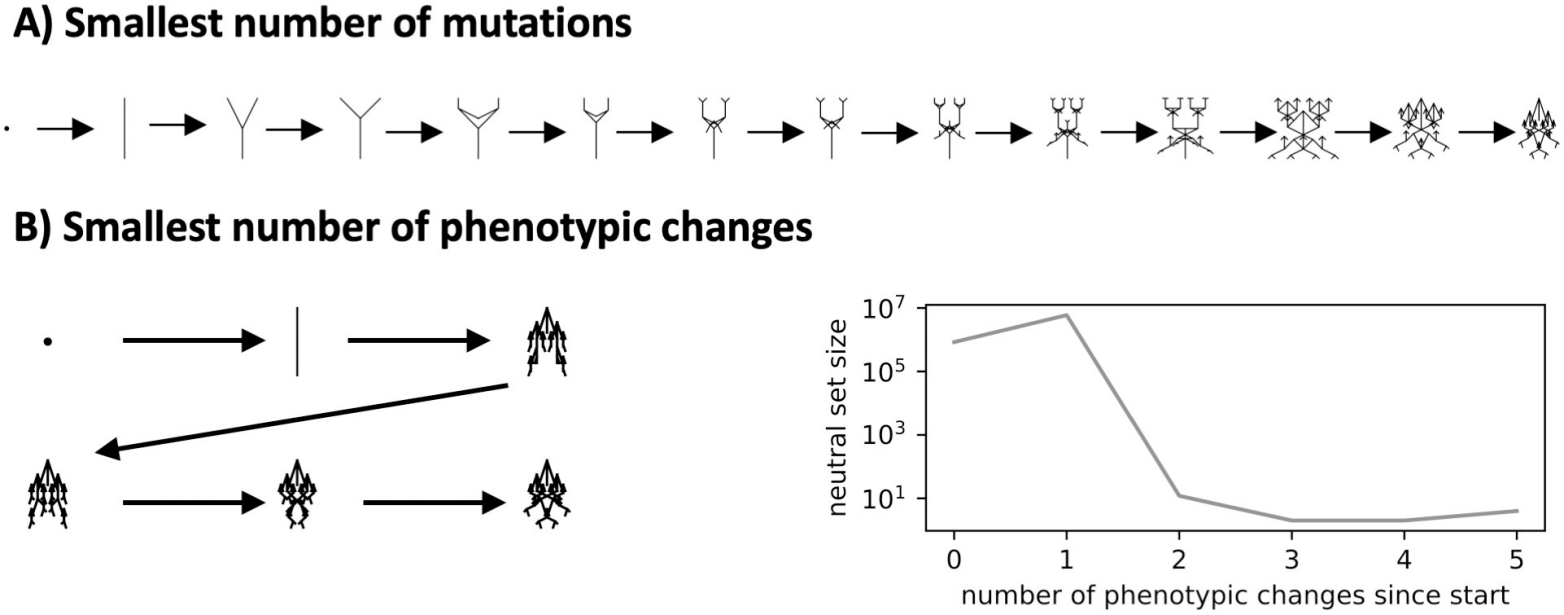
Reconstructing Dawkins’s search for a rare beetle-shaped phenotype. A) An evolutionary path from a dot to the final beetle-shaped biomorph that uses the smallest number of mutations from a given initial genotype that maps to a dot phenotype to the final beetle-shape phenotype. B) An evolutionary path from a dot to the final beetle shape with the smallest number of phenotypic changes (but several neutral mutations may be required between each of the phenotypic changes sketched in the figure). The neutral set sizes of the phenotypes along this path are shown on the right. A key advantage of this second scenario is that it increases the probability of a path without any fitness valleys for all intermediate steps (as recently demonstrated for molecular GP maps in Greenbury et al. [49]).

The previous subsection analyzed how the balance between selection and phenotype bias affects a single adaptive fixation step. In general, however, phenotypic adaptation is a multi-step process, and this is one of the key themes of *The Blind Watchmaker* [1]. Here we revisit one example which helps highlight the connection between multi-step paths in genotype space and fitness landscapes. In the book, Dawkins’s recounts how he had not recorded the genotype of an insect-shaped phenotype he had observed [1]. When he tried to find the insect-shaped phenotype again by artificial selection, this took a long time, even though he remembered what phenotypes were visited on the original evolutionary trajectory to the insect-shaped phenotype [1]. He explains the difficulty of finding the exact correct phenotype in terms of the shortest evolutionary paths between two phenotypes. Since Dawkins doesn’t write down the exact phenotype, we choose to pick one insect shape, a “beetle” (inspired by a biomorph example in ref [61]), and illustrate one of the paths with the smallest number of mutations in Fig 9A. To stay on this path, the ‘correct’ one out of 18 possible mutations (two possible changes for each of the nine genotype positions if we ignore boundary effects) has to be chosen at each step, so that the probability of obtaining this particular 13 step path is 1/18^13^ ≈ 5 × 10^−17^. Of course, there are many other paths that lead from the initial to the final genotype with the mutations arranged in a different order, so that the real probability of obtaining this phenotype by a random walk is closer to its phenotype frequency of *f*_*p*_ = 4/(7^8^ × 8) ≈ 9 × 10^−8^. Clearly, the probability of obtaining the final beetle phenotype by random mutations is extremely small [1]. By contrast, as illustrated by Dawkins’s second infinite monkey example [1], if there is a fitness function that allows each correct intermediate step to increase fitness, then the probability of success can become exponentially larger. Dawkins’s uses this example to argue that selection by many small steps is much more efficient at finding a fitness maximum than a naive mutationist picture where the final biomorph shape appears directly in a population [1]. One weakness of this example, and one shared schematically by his WEASEL program, is that it relies on a fitness function that is uphill for a large number of intermediate phenotypes.

The GP map perspective allows us to study a different kind of minimal path that explicitly includes neutral mutations, and which may facilitate stepwise evolutionary adaptation. Neutral mutations enable genetic drift and cryptic variation [62, 63]. These can facilitate adaptation because, although each genotype in a neutral set maps to the same phenotype, different genotypes will have different sets of accessible alternate phenotypes in their one-mutation neighborhoods [32]. With enough time, a population can in principle explore the entire neutral space, and so find all accessible phenotypes, the number of which is captured by *e*_*p*_, Wagner’s [32] measure of phenotype evolvability (See Fig 5D). Strictly speaking, not all genotypes in a neutral set are connected by neutral mutations and Wagner’s [32] phenotype evolvability needs to be computed for each mutationally connected subset, i.e. each neutral component [59]. Nevertheless, in this context, rather than ask what the absolute minimal number of mutations is, the more relevant question may be what minimum number of phenotypic changes a population has to pass through in order to evolve from a dot to a beetle. We illustrate an example of such a path in Fig 9B. Allowing the exploration of neutral networks greatly reduces the number of phenotypic transitions from a dot to a beetle when compared to the absolute shortest path shown in Fig 9A. Importantly, such pathways make it much easier to imagine how fitness could increase for all steps since the number of intermediate phenotypes is smaller. This scenario illustrates how neutral correlations in the GP map permit neutral exploration, which may facilitate the emergence of advantageous phenotypic transitions [32, 49]. In this example, concepts related to both the second and the third versions of the infinite monkey theorem defined above interact synergistically. While it is important to note that the exact length and type of the possible shortest phenotypic paths will depend sensitively on the coarse-graining, just as we saw before for phenotype evolvability values, our argument holds as long as phenotypic evolvabilities are higher than genotypic evolvabilities, since this ensures that exploring neutral spaces enables a higher number of transitions than are possible from a single genotype. This condition is met even in the fine-grained analytic model (see Fig 5).

## Discussion

### The biomorphs GP map shows many similarities to molecular GP maps

GP maps quantify exactly how random mutations get translated into a highly anisotropic exploration of the morphospace of phenotypes [3, 9, 64]. A key message of this paper is that the biomorphs GP map exhibits many qualitative similarities to molecular GP maps [7, 31]. The main similarities observed are listed below:

1. Biomorphs exhibit a strong phenotype bias: upon random sampling of genotypes, certain phenotypes are orders of magnitude more likely to appear than others. However, for biomorphs, a larger fraction of the morphospace of all structures have small neutral sets than is typically seen for molecular GP maps.
2. The particular form of the phenotype bias in biomorphs is typically towards phenotypes with short descriptions. Such ‘simplicity bias’ [3, 5] means that high-frequency phenotypes have low descriptional complexity, and only low-frequency phenotypes can have high descriptional complexity.
3. The mutational phenotype robustness *ρ*_*p*_ scales as the log of the frequency *f*_*p*_ that a phenotype is obtained upon random sampling of genotypes, and so is much higher than in a random null model without correlations between genotypes.
4. The mutational robustness ${\tilde{\rho }}_{g}$ of an individual genotype *g* is negatively correlated with a measure of its evolvability ${\tilde{\epsilon }}_{g}$ that counts the number of alternate phenotypes within a one-mutation neighborhood.
5. By contrast, the mutational phenotype robustness *ρ*_*p*_, calculated by averaging ${\tilde{\rho }}_{g}$ over the neutral set of *p*, is positively correlated with the phenotype evolvability *ϵ*_*p*_, which counts the number of different phenotypes accessible from the neutral set of all genotypes mapping to phenotype *p*.
6. The probability of non-neutral mutations *ϕ*_*pq*_ tends to increase with increasing frequency *f*_*p*_ of the target phenotype *p*, (if the initial phenotype *q* has a large enough neutral set). However, compared to molecular GP maps [40], biomorphs have an unusually high number of disallowed mutational links between phenotypes, so the positive correlation only holds for the small fraction of phenotypes that are linked by point mutations.
7. The relationships above can be analytically derived from a simple model that partitions the genomes into constrained regions that affect the phenotype and unconstrained regions that do not, but continue to hold in the absence of sequence-constraint effects for a constant gene nine.
8. The many orders of magnitude difference in the rate at which variation arrives in a population can lead to ‘arrival-of-the-frequent’ scenarios [14, 15] where a more frequent, but only moderately fit phenotype will fix in a population because the fitter phenotype either does not appear at all within the relevant time scales, or appears with too low a rate to have a meaningful probability of sweeping to fixation.
9. Neutral exploration can reduce the number of intermediate phenotypes needed to reach a fitness peak, increasing the likelihood that there are pathways that monotonically increase fitness.

The large number of similarities between biomorphs and molecular GP maps is at first sight surprising since the models have important qualitative differences. The molecular models most studied in the literature are typically based on minimum-free-energy folding (for example protein lattice models [65] and RNA folding models [66]), molecular self-assembly (for example models of protein quaternary structure [45, 67, 68]) or network topologies (for example gene regulatory networks [25]). By contrast, the biomorphs model’s organization is quite different. It imitates biological development through recursive local branching patterns [1].

Our analytic calculations reveal one key reason for the similarities between the GP map of biomorphs with that of molecular structures: the analytic model falls into the same class of sequence-constraint-based models that have been used to explain universal behavior observed in molecular GP maps [33, 34, 36]. However, sequence-constraint-based models are always just a simplification to a real GP map. In RNA, positions are often neither fully constrained nor fully unconstrained, but have variable constraints within a NC (see e.g. [69]). The same is true for the biomorphs GP map: when we restrict the value of the ninth gene to a constant (section E in S1 Text), the sequence constraints in the analytic model are the same for every genotype and phenotype, but the computational data still displays variations in neutral set sizes, complexities, robustness and evolvability. Under what conditions sequence-constraint-based provide a useful first approximation and whether these conditions hold in specific GP maps, is a topic for future research.

### Simplicity bias and increasing complexity in evolutionary history

Our simulations show that phenotypic bias can have an influence on adaptive evolution: when several phenotypes convey an adaptive advantage, the more frequent (and therefore usually the simpler) phenotype is more likely to evolve. It is important to note that this result is not in contradiction to arguments that complexity can increase over evolutionary time, see e.g. [70]. First of all, natural selection may simply favour more complex phenotypes. Secondly, there are normally many more phenotypes with higher complexities than those with lower complexities. Even if the probability that a *particular* individual phenotype appears upon random mutations is typically higher if its complexity is lower, the probability *P*(*K*) that a random mutation generates a phenotype of complexity *K* may still peak at a higher *K* because there are simply many more possible phenotypes with higher K (see [3] and section D.4 in S1 Text). Indeed, *P*(*K*) is a very broad distribution for biomorphs. Nevertheless, the question of how simplicity bias interacts with changes in morphological complexity over evolutionary time needs further study.

### Simplicity bias in developmental systems

If simplicity bias in GP maps follows from very general intuitions based on the algorithmic infinite monkey theorem, as formalised by AIT, then we might expect it to hold for a much wider range of GP maps than have been studied so far [3, 5]. Indeed, our results show that simplicity bias is observed beyond the molecular scale in Richard Dawkins’s biomorphs, which were created as a simplified description of morphological development. This then prompts the question of whether we should expect to see simplicity bias more generally in development.

Finding clear evidence for phenotype bias more generally, or simplicity bias more specifically, in developmental systems will be harder than for molecular systems. Problems typically studied in evo-devo are far from being as tractable or having the abundant data that the GP maps for RNA secondary structures or protein complexes have. What kind of evidence would one expect to find if simplicity bias is at play? One example where it has been invoked as a non-adaptive explanation is for the prevalence of high symmetry protein complexes [3]. The basic idea is easy to understand from the algorithmic picture of evolution. Less information is required to describe bonding patterns that lead to higher symmetry, and thus such phenotypes have a higher probability of appearing upon random mutations [3]. One could imagine extending this preference for symmetry, modulated by processes such as symmetry breaking [71], to larger-scale developmental processes (see [72, 73] for a discussion). In other cases, including the RNA secondary structures and branching morphologies (see ref [74]), different signatures of simplicity need to be employed to identify processes that can be described by shorter algorithms, which should be easier to find through random mutations. An alternative way of testing for simplicity bias would be to analyse if random mutations lead to simpler structures. Indeed, phenotypic changes observed in phylogenies of angiosperm leaf shapes [75] tend to be strongly biased towards simpler phenotypes and experiments on developmental pathways for mouse teeth suggest that mutations leading to simpler tooth shapes are more common than those that lead to increased tooth complexity because the latter scenario requires a coordinated change in several pathways [76]. Similar bias towards simplicity is also discussed in a recent study on the morphology of shark teeth [77].

### Future work for the biomorphs model

In his work on evolvability, Dawkins’s used the biomorphs “*as a generator of insight in our understanding of real life*” [29]. We believe that this tractable toy model of development has been understudied in the literature, and show that the biomorphs GP map displays a remarkably rich structure in the mapping from genotypes to phenotypes. These discoveries suggest a number of new directions in which our work on biomorphs could be extended. First of all, for computational efficiency, we only used a specific version of the model with nine genes, the same number that Dawkins’s used in *The Blind Watchmaker*. But the number of genes can be expanded, and several of the rules can be adapted [29]. Such changes to the genotype structure and the phenotype construction can allow the model itself to evolve, in other words, future simulations should not just model evolution *on* the GP map, but also evolution *of* the GP map, as advocated in ref [31]. With such an approach, one could study Dawkins’s formulation of the evolution of evolvability quantitatively and link it to some of the other ways that the concept evolvability is used (e.g. [52]). For example, certain types of structure in the arrival of variation may facilitate the evolution of phenotypic novelty [78–80]. Such changes to GP maps are likely candidates for being under positive selection, and biomorphs may form a good model system to investigate some of these proposals. These investigations could be supplemented with a second toy model introduced by Dawkins’s, the arthromorphs from his book “Climbing Mount Improbable” [81]. The arthromorphs produce a range of segmented 2D body plans inspired by arthropods such as Derocheilocaris [81].

By contrast to the RNA model, where the exact identity of the mutations is clear, in the biomorphs model, the mutations act on parameters and do not have as clear a biological identification. This more coarse-grained approach presents a challenge for modeling developmental systems [62]. Nevertheless, schematic models such as the biomorphs model have a long track record of success in evo-devo. Perhaps the most famous are growth models that have successfully been used to study developmental bias in plants [82]. Interestingly, gene-regulatory networks may also generically exhibit simplicity bias [25] and can display arrival-of-the-frequent like phenomena [83, 84]. Further work is needed to connect the results of schematic models to the underlying gene-regulatory networks.

Another direction for future research would be to look at the likelihood of phenotypic transitions (*ϕ*_*pq*_) in more detail. We found that transitions to high-neutral set size phenotypes tend to be among the most likely transitions, but also that many transitions are not possible in a single mutation so that *ϕ*_*pq*_ = 0. Future work could investigate whether these impossible phenotypic changes correlate with larger visual changes than possible phenotypic changes do. Recent arguments from AIT [85] predict that phenotypes with smaller conditional complexity $\tilde{K}\left(p|q\right)$ (e.g. phenotypes that are more similar to one another) are more likely to be connected by mutations. It is reasonable to expect that a mutation-induced change between more similar phenotypes will result in smaller fitness differences, lowering the probability of deleterious mutations, and increasing the likelihood of finding pathways with small incremental changes. Such correlations between the likelihood of phenotypic changes and their fitness are essentially what Dawkins’s exploited in the artificial selection experiments in The Blind Watchmaker [1]. By making incremental changes, he was able to evolve rare high-complexity structures such as his insect-shaped phenotypes. It would be interesting to study in more quantitative detail the interplay of random mutations and these phenotypic correlations on incremental adaptive evolution for biomorphs. This research program would entail combining the power of natural selection, demonstrated by Dawkins’s 2^nd^ infinite monkey theorem, with an algorithmic account of how structured variation arises, illustrated by the 3^rd^ monkey theorem. Such an interplay can help illustrate that phenomena such as developmental bias and natural selection are not in opposition, but should rather be seen as dual causes in a richer explanatory landscape. We believe that taking both creative forces into account should be far from “boring” [86]. Instead, their interaction opens up exciting new avenues for understanding how the remarkable power of evolution generates “*endless forms most beautiful*” [13].

## Supporting information

S1 Text Supplementary information as a combined text file. (PDF)
