# Supplementary material for: Bias in the arrival of variation can dominate over natural selection in Richard Dawkins’s biomorphs
Source: PLoS Comput Biol. 2024 Mar 27;20(3):e1011893. doi: 10.1371/journal.pcbi.1011893 (PMC10971585; doi:10.1371/journal.pcbi.1011893)
Supplement: S1 Text — (PDF) [file pcbi.1011893.s001.pdf]

Supplementary Information: Bias in the arrival of variation can dominate over natural selection in Richard Dawkins' biomorphs

Nora S. Martin, Chico Q. Camargo, Ard A. Louis

Contents

A Analytic constrained-unconstrained model 2
A.1 Neutral set sizes 2
A.2 Neutral set sizes and rank 3
A.3 Complexity estimates 4
A.4 Genotype and phenotype robustness 4
A.5 Genotype evolvability 5
A.6 Phenotype evolvability 5
A.7 Mutation probabilities 6

B Shape space covering property 6

C Robustness of GP map properties to changes in the phenotype definition 8
C.1 Grid size 8
C.2 Discretization 8
C.3 Analytic model results for different range of allowed genotypes 14

D Additional data on phenotype complexities 15
D.1 Examples of phenotypes and their estimated complexities 15
D.2 Alternative method of estimating phenotypic complexity 15
D.3 Alternative method of estimating phenotypic complexity in the analytic model 15
D.4 Distribution of phenotypic complexities for arbitrary genotypes 16
D.5 Simplicity bias in mutation probabilities 20

E GP map properties with a fixed number of developmental stages 21

F Identifying an evolutionary path with the smallest number of phenotypic changes 26

G Two-peaked landscapes - beyond the first fixation 26

H Selection for tree-like shapes 27

A Analytic constrained-unconstrained model

In the biomorphs model [1, 2], each integer in the genotype either affects the vectors from which the figure is built or the number of developmental stages after which the developmental process terminates, as illustrated in Fig 2 in the main text. The assumption we make when modelling the GP map analytically is that a mutation changes the phenotype if and only if it either affects a vector that is used in the final figure or if it changes the number of developmental stages. As discussed in the main text, this is accurate in an extremely detailed representation of the biomorphs, where the biomorphs are drawn with a fixed length scale on a very large high-resolution screen, lines that are generated multiple times in the developmental process are drawn as thicker lines and length-zero lines are somehow represented in the figure, for example as a dot. Otherwise, our assumption is simply a well-motivated approximation that we will use in the following to determine analytically whether two arbitrary genotypes share the same phenotype.

With this ansatz, we can build an analytic model similar to previous analytic GP map models, which rely on a division of sequences into constrained and unconstrained parts (for example in [3–5]): for a given phenotype, there are some positions in the genotype, where any mutation leads to a phenotypic change (constrained positions). Other positions can mutate without changing the phenotype (fully unconstrained positions). These definitions allow us to investigate the GP map analytically.

In our analytic treatment of the biomorphs GP map, we have the following division into constrained and unconstrained parts: g_9 is always constrained since mutations in the value of g_9 change the number of recursions, or developmental stages, and thus always lead to a phenotypic change. Since g_9 is constrained for all phenotypes, all genotypes in a given neutral set have the same value of g_9. The remaining eight genes, g_1 - g_8, which define the vectors in the biomorphs construction process, are constrained only for some phenotypes: whether the vector(s) encoded by a certain genotype position g_i appear in the final figure, depends on the value of g_9. If the vector(s) appear in the figure, any mutation to g_i changes the phenotype, and g_i is fully constrained. If the vector(s) do not appear in the figure, mutations to g_i have no effect on the phenotype in the analytic model and g_i is fully unconstrained. Thus, we can deduce the number of constrained positions n_u by studying Fig 2 in the main text and counting, how many genotype positions only appear in vectors that are not used in the final figure. This only depends on the number of developmental stages and thus on the value of g_9 in the neutral set of the given phenotype:

$$n_u(g_9) = \begin{cases} 9 - 2 \times g_9 & \text{if } 1 \leq g_9 \leq 4 \\ 0 & \text{if } 5 \leq g_9 \end{cases} \tag{1}$$

Thus, g_9 sets the number of unconstrained positions in a genotype and plays a similar role to the 'stop codon' in existing analytic constrained-unconstrained models (for example refs [3, 5]).

A.1 Neutral set sizes

The neutral set size can be computed following ref [4] if we know the number of unconstrained positions: in the neutral set of a given phenotype, each constrained genotype position is the same for all genotypes and each unconstrained position can take on any value. In our case, the values in the 'vector' part of the genotype

are restricted to integers between -3 and 3, so unconstrained positions can take one of $k = 7$ values. Thus, there are $k^{n_u(g_9)}$ possible sequences for the unconstrained part of the genotype.
Hence, there are $k^{n_u(g_9)}$ different genotypes that give the same phenotype, based on the constrained-unconstrained calculations. In addition, the biomorphs system has an axial symmetry that applies even to the constrained parts of the genotype[1]: flipping all x-coordinates in the figure does not change the phenotype. We approximate this by including a factor of two in our neutral set size estimates. This is only an approximation since the factor of two should not be applied if a genotype had zeros at all x-coordinates, but it gives the following simple expression for the neutral set size $N_p(g_9)$:

$$N_p(g_9) \approx 2k^{n_u(g_9)} \approx \begin{cases} 2k^{9-2\times g_9} & \text{if } 1 \leq g_9 \leq 4 \\ 2 & \text{if } 5 \leq g_9 \end{cases} \tag{2}$$

A.2 Neutral set sizes and rank

For our plot in the main text, we also need to compute the rank, i.e. the number of phenotypes with greater or equal neutral set size. We can deduce the rank as follows: since neutral set size decreases monotonically with g_9 (eq 2), we can express the rank as a sum over g_9. Since there are k^8 different genotypes for each fixed value of g_9 and $N_p(g_9)$ genotypes per phenotype, there should be $k^8/N_p(g_9)$ different phenotypes for a fixed value of g_9. With this, we can simply sum over all values of g_9 with greater or equal neutral set size to compute the rank. Because the neutral set size is the same for all $5 \leq g_9$, we need to handle this case separately:

$$r(g_9) = \begin{cases} \sum_{h=1}^{g_9} k^8/N_p(h) & \text{if } 1 \leq g_9 \leq 4 \\ \sum_{h=1}^{4} k^8/N_p(h) + (b-4) \times k^8/N_p(5) & \text{if } 5 \leq g_9 \end{cases} \tag{3}$$

Here, b is the number of different values g_9 can take (we assume that one is the lowest allowed value for g_9): in our case, g_9 can take any value from 1 to 8, so we have $b = 8$ and $b - 4 = 4$.
We can simplify the rank calculation in eq 3 by noting that the terms with the smallest neutral set sizes dominate the sums and putting in the expressions for N_p from eq 2. This means that we can approximate the full expression as:

$$r(N_p) \approx \begin{cases} k^8/N_p & \text{if } N_p > 2 \\ \frac{(b-4)}{2} \times k^8 & \text{if } N_p = 2 \end{cases} \tag{4}$$

Thus, for a range of neutral set sizes, the rank is proportional to N_p^{-1}, and conversely, the frequencies are proportional to $1/r(N_p)$ and so the distribution follows Zipf's law. This relationship is reminiscent of the power laws found in other GP maps, such as the Fibonacci model [3], for which the constrained/unconstrained approach was first developed. However, note that while eq 3 is exact for the analytic model, the reductions of the sums to their largest terms, which gave eq 4, are only an approximation that will lead to underestimates of the true sums, and thus the true ranks.

[1]In the context of previous models based on constrained/unconstrained approaches, this is equivalent to having multiple neutral components (NCs) per neutral set, for example in ref [6]: in that case, the constrained-unconstrained calculations give the size of a single connected NC, but the final result has to be multiplied by the total number of NCs to obtain the neutral set size.

A.3 Complexity estimates

In our analytic calculations, we do not draw the biomorphs figures in 2D and so it is not possible to estimate their complexities from drawn images. However, it is possible to derive an upper bound on the complexity of a biomorph phenotype without drawing the corresponding figure as follows: one way of describing a biomorph phenotype is by recording one corresponding genotype as well as the instructions on how to generate the figure from the genotype. As in previous applications of AIT arguments to GP maps [7], we will ignore the second part, which is a constant term that is the same for all phenotypes. The genotypes, however, are different for different phenotypes: since only the constrained genotype positions are required to fully define the phenotype, the length of the essential part of the genotype varies from phenotype to phenotype. The length of this essential part is proportional to the number of constrained positions per genotype. This is one upper bound on the complexity since the complexity is defined as the shortest possible description length and the genotype is one way of describing the phenotype. Thus, we have an upper bound on complexity $\tilde{K}$ as:

$$\tilde{K} \leq a \times (9 - n_u(g_9)) \tag{5}$$

where a is the constant of proportionality that is set by the description length per encoded phenotype position. In our analysis, sites g_1 to g_8 can take one of seven discrete values and g_9 can take one of eight discrete values - thus any genotype position can be encoded in $a = \log_2 8 = 3$ bits. Using eq 2 to express n_u in terms of neutral set sizes then gives a log-linear upper bound:

$$\tilde{K} \leq 3 \times (9 - \log_2(N_p/2)/\log_2(k)) \tag{6}$$

Rearranging for N_p gives:

$$N_p \leq 2k \times 2^{9-\tilde{K}/3} \tag{7}$$

A.4 Genotype and phenotype robustness

In order to find the robustness of a genotype ρ_g, we need to compute the fraction of mutations that leave its phenotype unchanged. By definition, all mutations at unconstrained sites leave the phenotype intact, whereas all mutations at constrained sites change the phenotype. Thus, we only need to know the number of constrained sites and the number of possible mutations at each site. The number of possible mutations at each site is two (changing the value at the site by +1 or -1) if we neglect the fact that in our model, the values are confined to fixed ranges (for example -3 is the lowest value g_1 can take and cannot be decreased further). With this assumption, we have:

$$\rho_g \approx 2/18 \times n_u(g_9) \tag{8}$$

This only depends on g_9 and all genotypes in the neutral set of a phenotype share a single value of g_9, all genotypes in a neutral set have the same robustness. Thus, the average robustness of a phenotype is simply:

$$\rho_p \approx 1/9 \times n_u(g_9) \tag{9}$$

So far, we have written phenotypic robustness ρ_p as a function of g_9. We can rewrite this as a function of phenotypic frequency using eq 2 and relying on the fact that the phenotypic frequency is simply the neutral set size normalized by the total number of genotypes, which is bk^8:

$$\rho_p \approx 1/9 \times \log_k(N_p/2) = 1/9 \times \log_k(k^8 \times b \times f_p/2) \tag{10}$$

A.5 Genotype evolvability

If we start with a given initial genotype, each distinct mutation in a constrained site changes the phenotype in a distinct way and so there are $\widetilde{\epsilon}_g \approx 2 \times (9 - n_u(g_9))$ distinct phenotypic changes (again assuming there are always exactly two mutations at each site). Using Eq 9, we have the following relationship between genotype robustness and evolvability:

$$\widetilde{\epsilon}_g \approx 18 \times (1 - \rho_g) \tag{11}$$

A.6 Phenotype evolvability

Calculating the phenotype evolvability is a little more complex: for this, we need to compute how many distinct phenotypic changes are possible from the entire neutral set of the initial phenotype p, a task similar to calculations in ref [5]. This can be higher than the evolvability of an individual genotype if different phenotypic changes are possible for different genotypes in the neutral set of p. In our biomorphs model, this is the case for mutations that raise g_9 by $+1$: since such a mutation adds one recursion in the construction process, the mutation can cause additional vectors to appear in the figure. This means that there can be sites, which were unconstrained before the mutation, but play a determining role for the phenotype after the mutation. Thus, a single mutation - raising g_9 by $+1$ - can generate several distinct phenotypic changes when applied to different genotypes in the neutral set of p.

The number of distinct phenotypic changes that can be achieved from a given initial phenotype by raising g_9 by $+1$ can thus be computed by identifying the number of sites that switch from being unconstrained to constrained, $(9 - n_u(g_9 + 1)) - (9 - n_u(g_9))$, and the number of values each of these sites could take, $k = 7$. Thus, we have $k^{n_u(g_9) - n_u(g_9+1)}$ distinct phenotypic changes that can be achieved from a given initial phenotype by raising g_9 by $+1$.

All other non-neutral mutations - lowering g_9 or changing a constrained 'vector component' site - have the same phenotypic effect regardless of which genotype in the neutral set of p they are applied to. The number of such non-neutral mutations is $2 \times (9 - n_u(g_9)) - 1$. Thus, we can add both contributions to obtain an expression for the phenotypic evolvability of p:

$$\epsilon_p \approx 2 \times (9 - n_u(g_9)) - 1 + k^{n_u(g_9) - n_u(g_9+1)} \tag{12}$$

The first term of this expression scales like the genotype evolvability and is anti-correlated with robustness. It is therefore the last term that gives us a positive correlation between robustness and evolvability on the phenotypic level. As postulated in previous theoretical work [5], this term is due to mutations that change the sequence constraints. This role corresponds to mutations in the 'stop codon' [5], and thus to mutations in g_9 in the biomorphs model.

So far, eq 12 is only a parametric equation. However, we can put in values of $1 \leq g_9 \leq 8$ and use equations 1 & 9 to get the following cases, depending on the value that $n_u(g_9) - n_u(g_9 + 1)$ can take:

$$\epsilon_p \approx \begin{cases} 18 & \text{if } \rho_p = 0 \\ 15 + k & \text{if } \rho_p = 1/9 \\ 18 \times (1 - \rho_p) - 1 + k^2 & \text{if } 2/9 \leq \rho_p \end{cases} \tag{13}$$

A.7 Mutation probabilities

Mutation probabilities ϕ_{pq} quantify the likelihood that a new phenotype p is generated by a random mutation applied to a random genotype in the neutral set of an initial phenotype q [8]. Both mutation probabilities and evolvability values capture phenotypic changes and thus our calculations of mutation probabilities ϕ_{pq} will use the same arguments as in the previous paragraphs: most mutations bring about the same phenotypic change for all genotypes in the neutral set. Phenotypic changes produced by such mutations are generated by one in every eighteen mutations since there are eighteen mutations for each genotype (again ignoring boundary effects) and only one mutation gives the specific phenotypic change. The one exception is again a mutation that raises the value of g_9: since there are $k^{n_u(g_9)-n_u(g_9+1)}$ different phenotypic changes produced by this type of mutation in a given initial neutral set, each of these occurs with $\phi_{pq} \approx 1/(18 \times k^{n_u(g_9)-n_u(g_9+1)})$. To sum up, the possible phenotypic changes have the following likelihood:

- Decreasing g_9 by one: this gives a new phenotype p with $\phi_{pq} \approx 1/18$. The neutral set size of p is given by $N_p(g_9 - 1)$.
- Increasing g_9 by one: this gives a new phenotype p with $\phi_{pq} \approx 1/(18 \times k^{n_u(g_9)-n_u(g_9+1)})$. The neutral set size of p is given by $N_p(g_9 + 1)$.
- Mutating any other non-neutral site: this gives a new phenotype p with $\phi_{pq} \approx 1/18$. The neutral set size of p is given by $N_p(g_9)$.

These three combinations of ϕ_{pq} and neutral set size give the analytic prediction plotted in the main text (where the initial phenotype has $g_9 = 3$). It is important to note that not all phenotypic changes are possible: even if q and p share the same value of g_9, there is no way of mutating from q to p if one of the constrained sites differs by ≥ 2 since constrained sites are constant in an entire neutral set by definition and can only change by ± 1 in a single non-neutral mutation. Thus, the analytic model predicts that most phenotypic changes cannot be achieved in a single mutation (i.e. $\phi_{pq} = 0$).

Thus, frequent phenotypic changes occur with $\phi_{pq} \approx 1/18$ and correspond to changes in the vector part of the genotype or to changes in g_9 that lower the number of developmental stages, i.e. broadly examples of heterometry or a specific type of heterochrony if we follow the classification of developmental changes in ref [9] (depending on how we assume that the developmental stages in the biomorphs system are controlled by gene-regulatory events). Rarer phenotypic changes occur with $\phi_{pq} \approx 1/(18 \times k^{n_u(g_9)-n_u(g_9+1)})$ and correspond to changes in g_9 that increase the number of developmental stages, i.e. to a specific type of heterochrony.

B Shape space covering property

In the main text, we performed many analyses that have been applied to a range of molecular GP maps. For completeness, we include one further aspect here, the concept of 'shape space covering': this concept postulates that when we start with a given initial genotype and consider all genotypes within a mutational distance of at most d from that genotype, then most high-frequency phenotypes exist among this set of genotypes, even when the value of d is small [10]. Other authors use a slightly different definition of 'shape space covering' that is not limited to high-frequency phenotypes, but includes all phenotypes [11]. However,

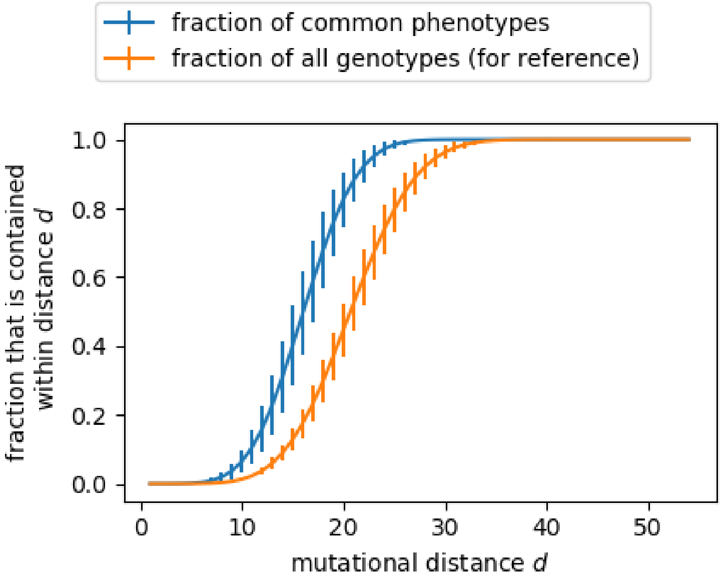

Figure A: ***Test of the shape space covering concept:*** *For ten randomly selected initial genotypes, all genotypes within a mutational distance of d are enumerated and their phenotypes recorded. We evaluate, what fraction of high-frequency phenotypes are found within this set for a given value of d (blue). For reference, we also show, what fraction of all genotypes are contained within this set (orange). The plot shows the mean and standard deviation of the values for the ten different initial genotypes. Note that the fraction of all genotypes that are contained within a distance d depends on the initial genotype due to our definition of the biomorphs' genotype space: if the initial value at a given site is* -3*, then it can take up to six mutations to reach every value in the valid range* $[-3, 3]$*, whereas this would only take up to three mutations if the initial value was* 0*.*

here we work with the original definition.

Here, we test the hypothesis of 'shape space covering' by following the methods in ref [10]: we start with a randomly selected genotype and evaluate the fraction of phenotypes found within at most d mutations from that genotype. This analysis is repeated for several initial genotypes and the results are shown in Fig A: we find that the number of high-frequency phenotypes covered within d mutational steps increases rapidly with d and that about 50% of these frequent phenotypes are found after around $d \approx 15$ mutations, even though the maximum distance between two genotypes is 55 if each integer value had to change from the lowest permitted value (-3 for $[g_1, ..., g_8]$ and 1 for g_9) to the highest permitted value (3 for $[g_1, ..., g_8]$ and 8 for g_9). In this analysis, we have followed the definition by Grüner et al. [10] and considered a phenotype to be among the high-frequency phenotypes if its phenotype frequency is higher than the average phenotype frequency of all phenotypes.

Two reasons are given in the literature for why the 'shape space covering' property is found in many GP maps: first, the set of genotypes within a mutational distance of at most d grows rapidly with mutational distance d due to the many ways in which d mutations can be combined along the genotype (i.e. because the

mutational space is high-dimensional) [12]. Secondly, high-frequency phenotypes are often so frequent that they are likely to be found among even a small set of random genotypes [13]. We can investigate the first aspect by recording the fraction of all valid biomorph genotypes that are found within d mutations of an initial genotype (orange line in Fig A). We find that this fraction increases quickly in only a few mutational steps, as expected. However, we also find that for a given d, the fraction of high-frequency phenotypes within d is even higher than the fraction of all genotypes. Therefore, the second argument also applies: high-frequency phenotypes are likely to be found in a relatively small set of genotypes, simply because there are many genotypes mapping to each of these phenotypes.

C Robustness of GP map properties to changes in the phenotype definition

In the methods section of the main text, we describe, how we convert each 2D biomorphs figure into a discrete phenotype for our computational analysis. This process relies on two parameters: the grid size and the threshold above which a pixel is set to one. In our analysis, we used a 30×30 grid and set a pixel to one if the total length of all unique line segments within that pixel equaled at least $\geq 20\%$ of the width/length of the pixel. In this section, we vary these two parameters and repeat key aspects of the GP map analysis, in order to test how robust our results are to the details of the phenotype definition.

C.1 Grid size

GP map data for different values of the grid size are shown in Figs B-C: a lower resolution of 20×20 is used in Fig B and a higher resolution of 40×40 is used in Fig C. We find that the qualitative results of the analysis are unchanged: we still find phenotypic bias over several orders of magnitude, this bias is towards a subset of the simple phenotypes, phenotype robustness is correlated with the logarithm of the neutral set size, mutation probabilities (if non-zero) tend to be higher for higher-frequency phenotypes and the relationship between robustness and evolvability is negative on a genotypic level, but (weakly) positive on a phenotypic level. All these results continue to be in agreement with the simple analytic model, and the simplicity bias remains consistent with the log-linear upper bound predicted by Dingle et al. [7].
While the qualitative trends and results are all robust to different parameter choices, the quantitative data does show differences: for example phenotypic evolvability values tend to be higher when using a more coarse-grained treatment on a 20x20 grid than when choosing a more fine-grained treatment on a 40x40 grid (Fig BF compared to Fig CF). This observation can be explained as follows: in a more coarse-grained treatment, more sequences belong to a given neutral set and thus neutral set sizes are higher. Such changes can have a big effect on evolvability since a single transition from p to a new phenotype q from a single genotype in the neutral set of p is sufficient to raise the evolvability by one for the entire neutral set.

C.2 Discretization

Similarly, the analysis was repeated for different values of the discretization threshold: this threshold t determines whether a pixel, which contains a number of line segments with a total length of l, is set to zero or one: it is set to one if $l \geq t$ and zero otherwise. Here, we repeat the GP map analysis with values of

10% of the pixel size (Fig D) and 50% of the pixel size (Fig E). Again, we find that the qualitative GP map characteristics, as well as the agreement with the analytic model and the predictions from [7], are unaffected.

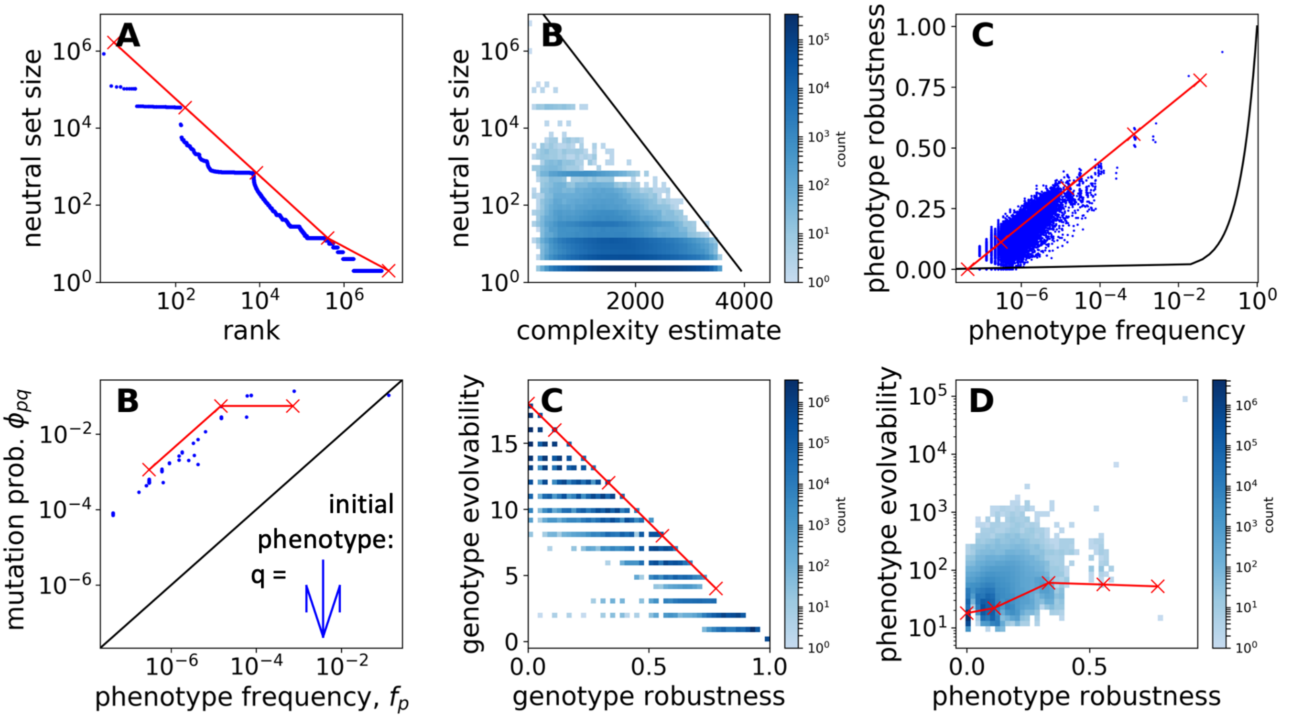

Figure B: ***GP map analysis with a different parameter choice in the coarse-grained phenotype definition:*** *Here, a* 20×20 *grid is used (instead of* 30×30*). The analysis shows the GP map data (blue) as well as the predictions from the analytic model (red) for the following quantities:* ***(A) Neutral set size vs frequency rank. (B) Neutral set size vs estimated complexity.*** *The black line indicates an approximate log-lin upper bound to guide the eye, as predicted in [7].* ***(C) Phenotype robustness vs phenotype frequency*** f_q***.*** *The black line (*$\rho_q = f_q$*) shows what we would expect in the null model from ref [14].* ***(D) Phenotype mutation probability*** ϕ_{pq} ***vs. phenotype frequency*** f_p ***for one specific initial phenotype*** q***.*** *The black line (*$\phi_{pq} = f_p$*) shows what we would expect in the null model from refs [8, 14]. Data points with* $\phi_{pq} = 0$ *are excluded on this log-scale.* ***(E) Genotype evolvability vs genotype robustness. (F) Phenotype evolvability vs phenotype robustness.*** *Only the computational data depends on the grid size; the analytic data is included just for reference.*

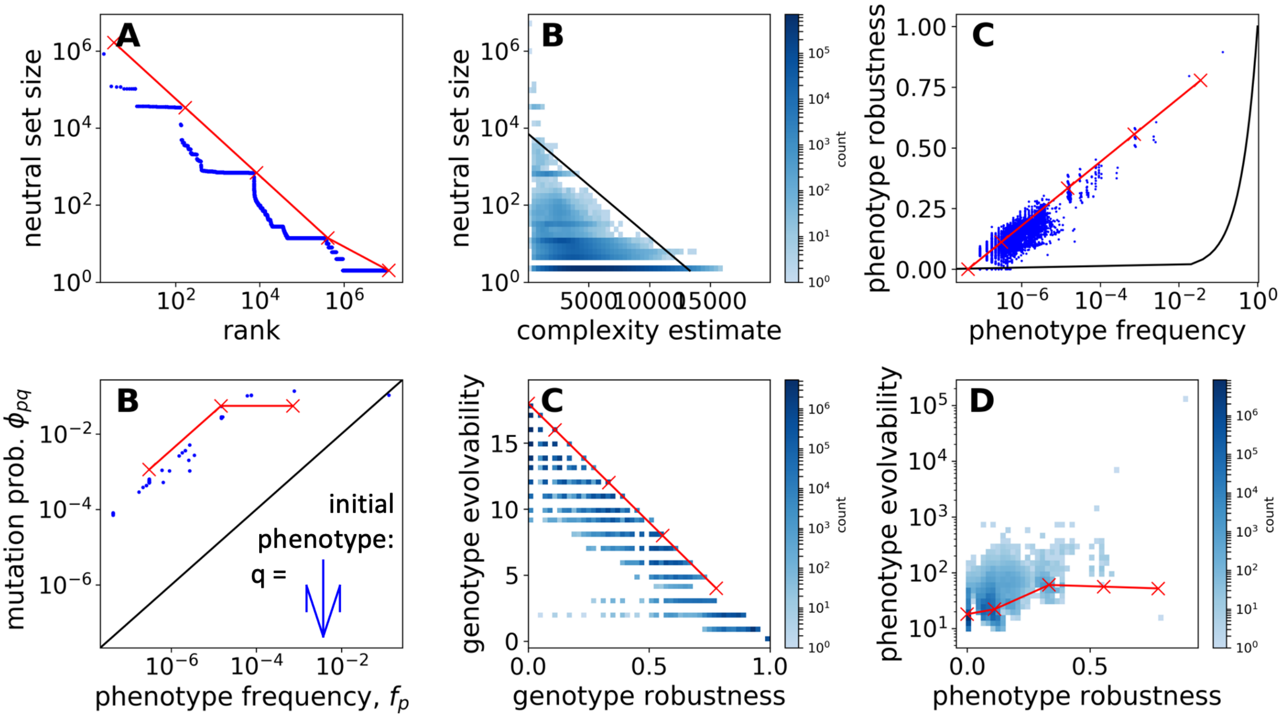

Figure C: ***GP map analysis with a different parameter choice in the coarse-grained phenotype definition:*** *same as Fig B, but here a* 40×40 *grid is used.*

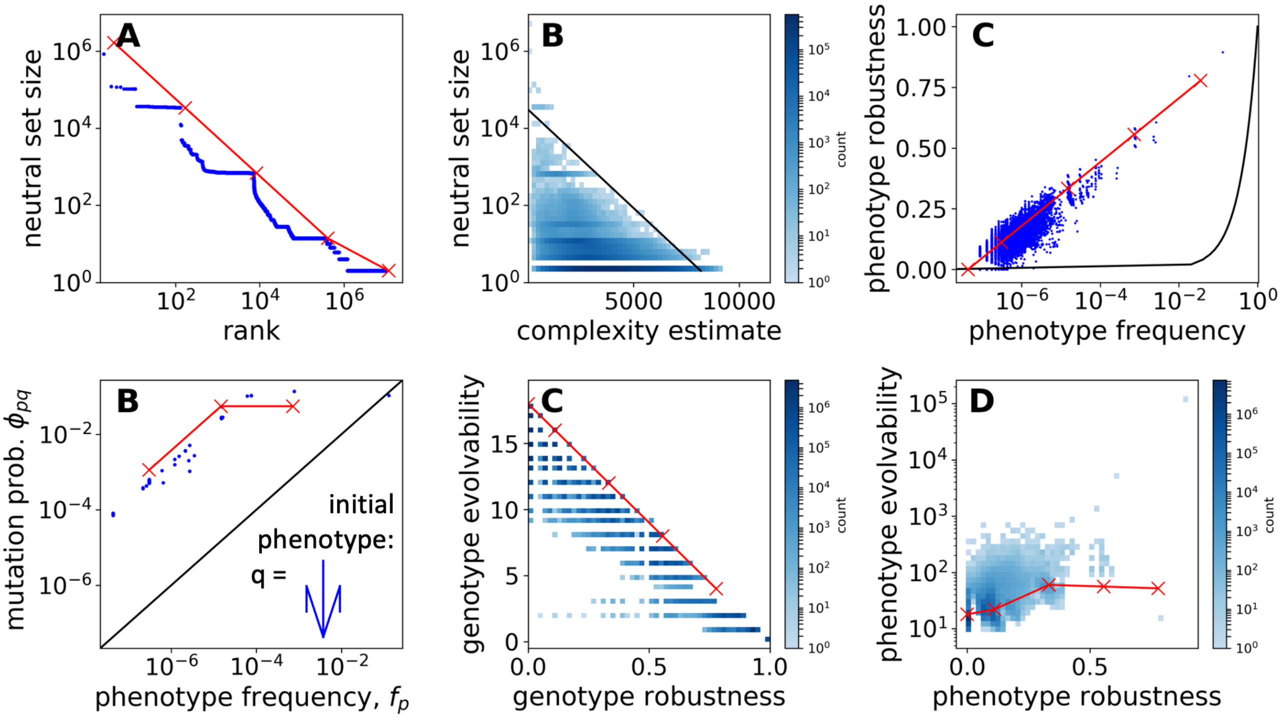

Figure D: ***GP map analysis with a different parameter choice in the coarse-grained phenotype definition:*** *same as Fig B, but here the threshold is different from the one in the main text:* 10% *instead of* 20%. *The grid size,* 30×30, *is the same as in the main text.*

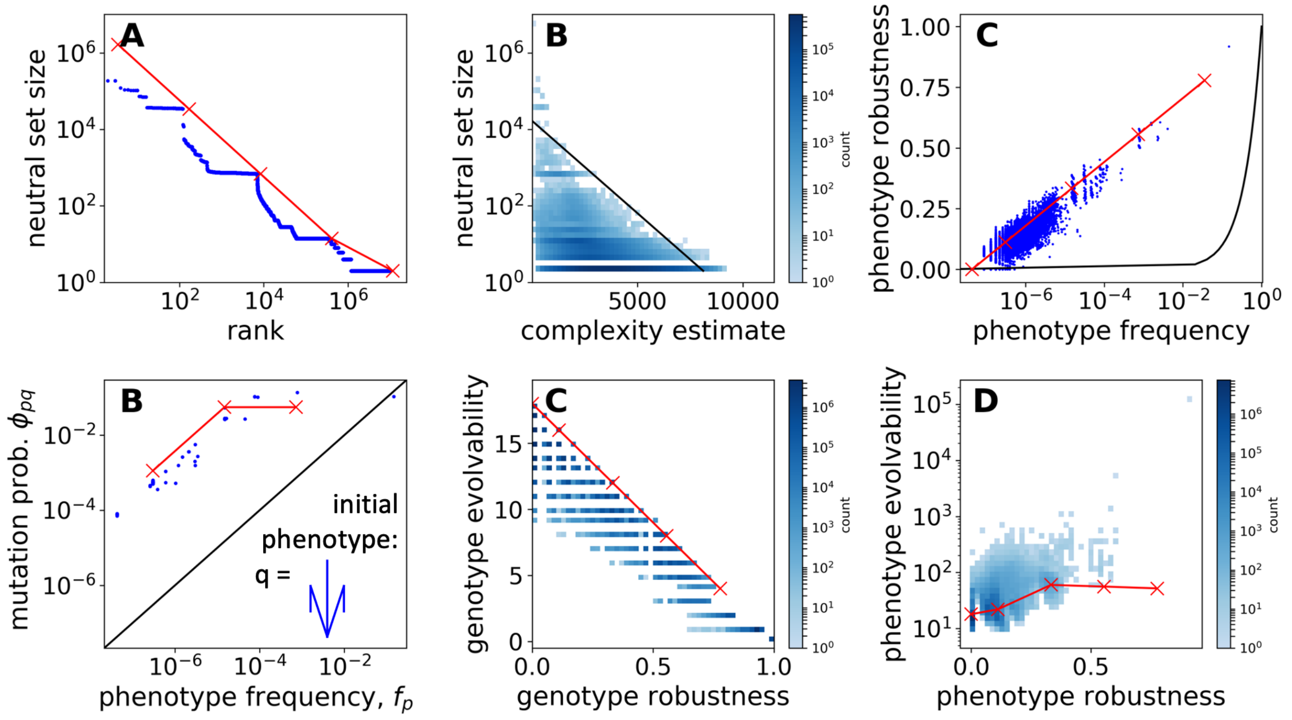

Figure E: ***GP map analysis with a different parameter choice in the coarse-grained phenotype definition:*** *same as Fig B, but here the threshold is different from the one in the main text:* 50% *instead of* 20%*. The grid size,* 30×30*, is the same as in the main text.*

C.3 Analytic model results for different range of allowed genotypes

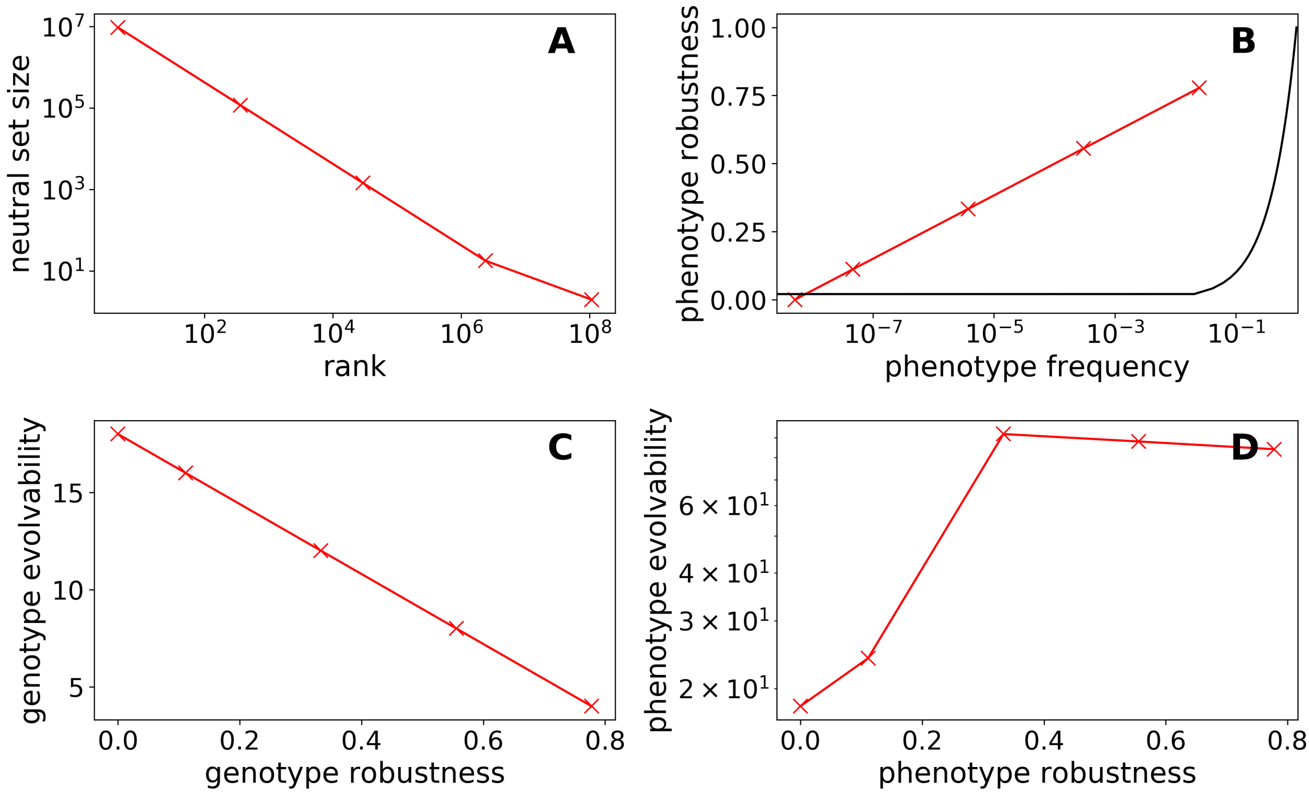

Figure F: ***Predictions from the simplified analytic model for a larger range of permitted values at each genotype position:*** *here, a larger range of values is permitted at each position of the genotypes than in the main text: nine values for each of the 'vector genes' ($-4 \leq g_i \leq 4$ for $i \in [1,..,8]$) and nine values for the ninth gene ($1 \leq g_9 \leq 9$). With these parameters, there are $9^9 \approx 4 \times 10^8$ genotypes and so a full computational analysis is no longer feasible. However, approximate predictions from the analytic model can be made and these are shown in this figure. The plots show: A) Neutral set size vs. frequency rank (Eq 4). B) Phenotype robustness vs. phenotype frequency (Eq 10). C) Genotype evolvability vs. genotype robustness (Eq 11). D) Phenotype evolvability vs. phenotype robustness (Eq 13).*

With our analytic model, we can allow an arbitrary range of values in the genotypes, without computational difficulties. Here we consider a GP map, where the genotypes can take on a wider range of values: nine values for each of the 'vector genes' ($-4 \leq g_i \leq 4$ for $i \in [1,..,8]$) and nine values for the ninth gene ($1 \leq g_9 \leq 9$). The data in Fig F indicate that permitting a wider range of integers in the genotype would not affect our qualitative results.

D Additional data on phenotype complexities

D.1 Examples of phenotypes and their estimated complexities

In order to visualize the data in Fig 4C of the main text, we focus on phenotypes with different combinations of complexity values and neutral set sizes: Fig G shows examples of simple phenotypes with low neutral set sizes, simple phenotypes with high neutral set sizes and complex phenotypes with low neutral set sizes (complex phenotypes with high neutral set sizes do not exist since we have observed simplicity bias in this GP map). While the analytic model cannot be compared directly to this data, which is based on the computational phenotype treatment, we can still use insights from the analytic model to guide our interpretation: we find that, as we might expect from the analytic model, phenotypes with few lines (and hence low g_9 and high neutral set sizes) are simple. Phenotypes with many lines (and hence high g_9 and low neutral set sizes) can have high complexity, but they can also have low complexity in the coarse-grained computational model, for example, if each vector is used exactly once or if some vectors overlap.

D.2 Alternative method of estimating phenotypic complexity

Since Kolmogorov complexity cannot be measured exactly, just estimated, we repeated the analysis in the main text for a different method of estimating complexity: we use a compression-based method, the Lempel-Ziv approach, relying on the implementation from ref [7]. This implementation takes a binary string as an input, but our phenotypes are 2D binary grids. Therefore, we concatenated the rows of our grid before passing it to the complexity estimator. Since Dingle et al. [7] argue that the complexity of a string is best estimated as the mean of the estimated complexity of the string and the estimated complexity of its reverse, we performed an analogous calculation on our 2D array: we took the transpose of the binary grid and included it in the estimate. This means that we did not only take a mean of the estimated complexity of the string and its reverse, but also the corresponding concatenated string of the transposed array and its reverse. The results from this compression-based complexity estimator are shown in Fig H: we still find that there are no complex phenotypes with high neutral set sizes. As before, the data approximately falls below a log-linear line, derived theoretically in ref [7].

D.3 Alternative method of estimating phenotypic complexity in the analytic model

In the analytic model, we have a clear criterion for when two genotypes fall into the same neutral sets, formulated in terms of constrained and unconstrained sites, but we do not have a visual description of each corresponding phenotype. Thus, we estimated phenotype complexities using the information that needs to be encoded in the genotype (section A.3). However, there is one way of approximating the visual complexity of each phenotype: if we simply assume that each line in a phenotype drawing takes the same amount of information to encode, then the number of lines in the biomorphs figure is a good proxy for the total description length, i.e. the complexity. This is only an approximation since a set of parallel lines can be encoded more efficiently than a set of arbitrary lines (in the same way that repeating strings can be compressed, but arbitrary strings cannot). However, if we use the number of lines n_l that are drawn in the biomorphs image, whether they are overlapping or not, as a first approximation, we get:

$$n_l = 2^{g_9} - 1 \tag{14}$$

Since we also have an expression for the neutral set size as a function of g_9 (eq 2), we can use this parametric relationship to obtain the data in Fig I. We find that the qualitative trend is the same: more complex phenotypes with a higher number of lines tend to have lower neutral set sizes. However, this relationship no longer follows the log-linear relationship predicted in ref [7]. This is because the number of lines in the biomorphs figure can exceed the number of distinct vectors (which is eight). Then the same vectors appear with different scaling factors multiple times in the figure and so the number of lines increases more rapidly than the amount of genotypic information needed to encode them.

D.4 Distribution of phenotypic complexities for arbitrary genotypes

In the main text, we found that a phenotype with a large neutral set is likely to be simple. Thus, a given simple phenotype is more likely to have a high phenotype frequency and appear in a small random sample of genotypes than a given complex phenotype. However, there may be many different simple phenotypes and many different complex phenotypes, so it is not clear, how many simple phenotypes we expect to find overall in a random sample of genotypes. Arguments in the SI of ref [16] imply that while the neutral set size of an individual complex phenotype is small, the number of distinct complex phenotypes is much larger than the number of distinct simple phenotypes, and so overall, the likelihood of drawing any complex phenotype from a random sample of genotypes is approximately equal to the likelihood of drawing any simple phenotype.

Here we test both parts of this argument for the biomorphs GP map, using our computational results. First, we consider the number of simple and complex phenotypes (Fig J): we find that there is only a small number of simple phenotypes, as expected from the information-theoretic arguments in ref [16] . However, the number of very complex phenotypes is also limited. This deviation from the information-theoretic arguments in ref [16] is likely due to the constraints of the biomorphs system, which only permits certain geometric forms (for example no phenotype can have two disconnected parts and this fact alone severely restricts the number of possible 2D drawings). Secondly, we calculate the fraction of *genotypes* with simple or complex phenotypes (Fig K). We find that there is a range of complexity values, for which the probability of finding a phenotype with that complexity is to first order constant, in agreement with the information-theoretic arguments in ref [16]. However, at high complexities, we see a deviation from the flat distribution expected from information-theoretic arguments, which is consistent with the deviations seen on the phenotypic level in Fig J.

So far, complexity distributions like this have not been discussed in much detail (exceptions are for example in the SI of ref [16], and for one matrix-rewriting grammar GP map in ref [17] and digital logic gates in ref [18]) and so future work should investigate these distributions and their implications more thoroughly, both for the biomorphs and for other GP maps.

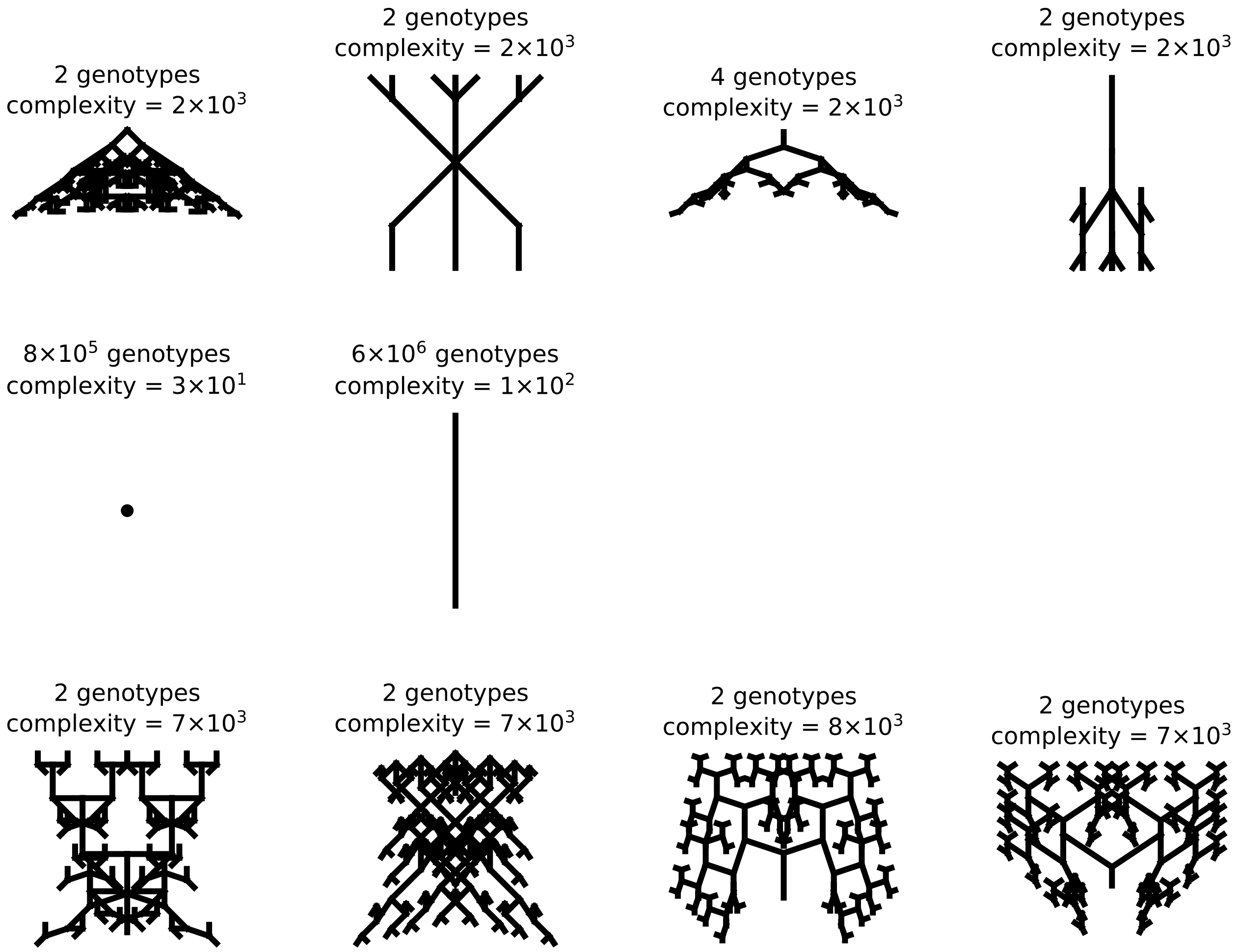

Figure G: ***Neutral set sizes and complexity estimates for a few example phenotypes:*** *the figures show examples of rare and simple phenotypes (top row), of frequent and simple phenotypes (middle row), and of rare and complex phenotypes (third row). Note that the labels 'rare'/'frequent' and 'complex'/'simple' are discrete categories that represent a range of values - not all of the 'simple' phenotypes have the same complexity in this figure.*

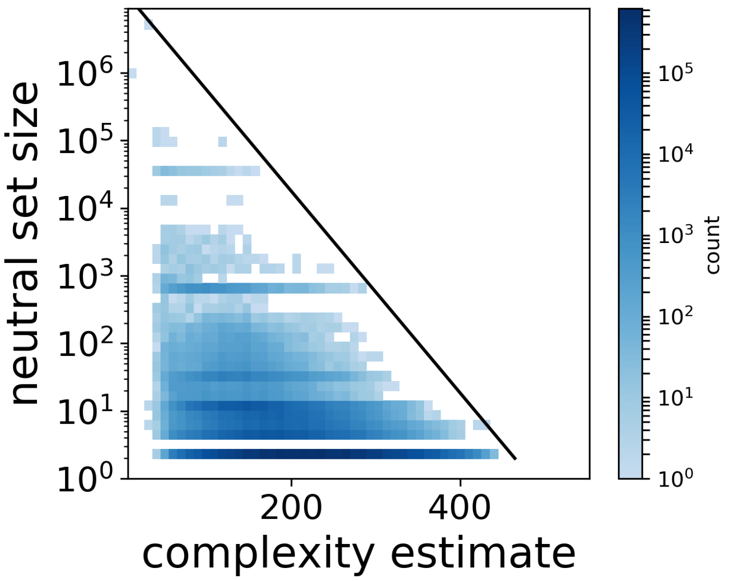

Figure H: ***Neutral set size vs complexity estimate with a compression-based complexity estimator:*** *as in Fig 4C in the main text, we plot the neutral set size of each phenotype (on a log scale) against an estimate of its complexity. Here, this complexity is computed by feeding a concatenated version of each phenotype's binary pixel array into the Lempel-Ziv compression implementation of ref [7]. A log-linear line, as predicted as an upper bound [7], is drawn to guide the eye.*

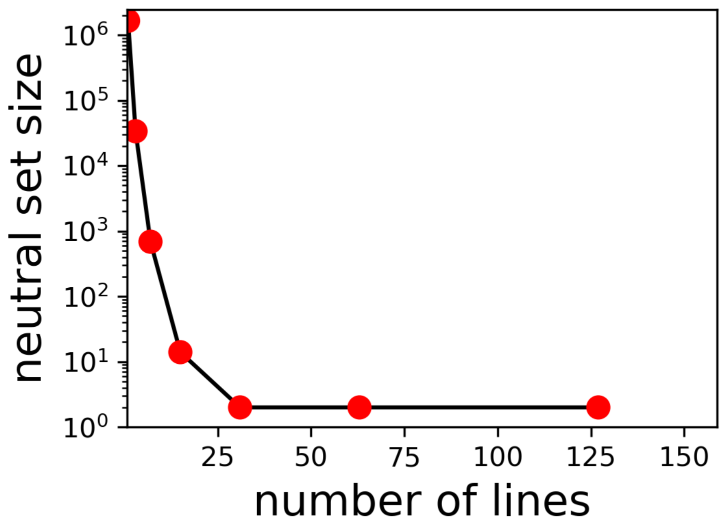

Figure I: ***Neutral set size vs number of lines in the figure:*** *both quantities are estimated with the analytic model, using eq 2 and eq 14.*

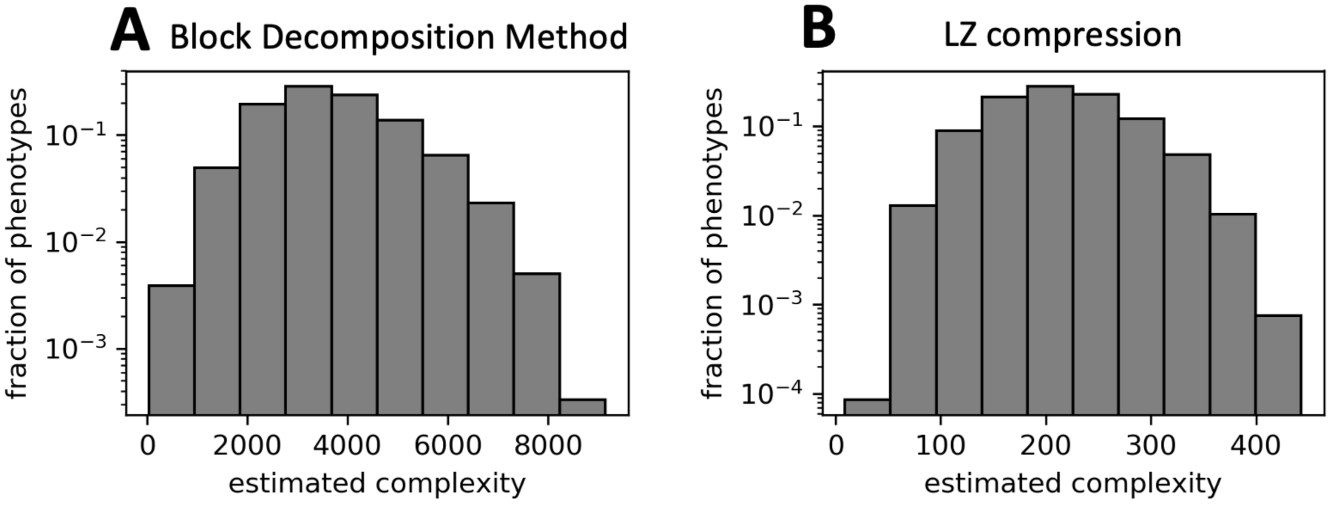

Figure J: ***Probability $\mathbf{P_p(K)}$ of obtaining a phenotype of complexity $\mathbf{K}$ upon random sampling of phenotypes.*** *Most of the $\approx 10^7$ phenotypes have intermediate complexity values - phenotypes with very low or very high complexities are rare. A) Phenotypic complexities based on the Block Decomposition Method [15] (as in the main text). B) Phenotypic complexities based on Lempel-Ziv compression (as in section D.2 above).*

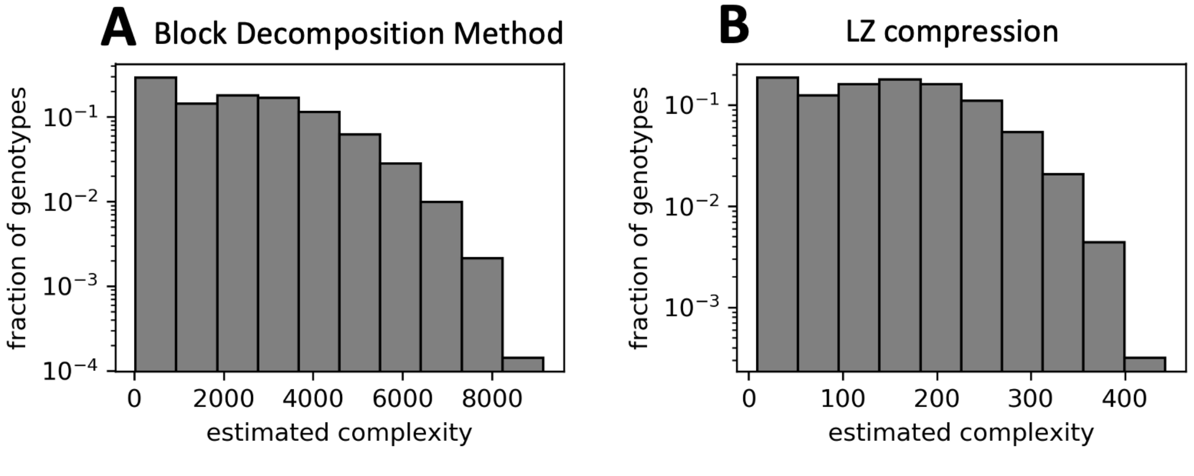

Figure K: ***Probability $\mathbf{P_g(K)}$ of obtaining a phenotype of complexity $\mathbf{K}$ upon random sampling of genotypes.*** *A) Phenotypic complexities based on the Block Decomposition Method [15] (as in the main text). B) Phenotypic complexities based on Lempel-Ziv compression (as in section D.2 above).*

D.5 Simplicity bias in mutation probabilities

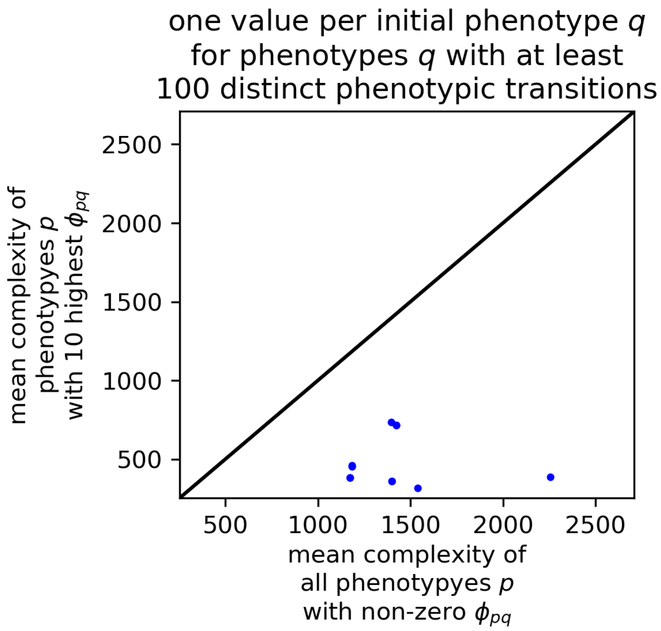

Figure L: ***Mutational bias towards simple phenotypes:*** *we consider all phenotypes q, from which at least 100 different phenotypes p can be reached through mutations (i.e.* $\epsilon_q \geq 100$*). For each initial phenotype q, we plot the mean complexity of the 10 phenotypes p with the highest* ϕ_{pq} *values against the mean complexity of all phenotypes with non-zero* ϕ_{pq} *values, in order to compare likely phenotypic transitions to the full set of possible phenotypic transitions. The black line indicates equality (*$x = y$*). We find that for all initial phenotypes q, the complexity of the high-*ϕ_{pq} *phenotypes is lower, indicating that mutation probabilities to simple phenotypes q tend to be higher. The data in this plot is for our computational approach based on the coarse-grained images.*

In the main text, we argued that the strong simplicity bias found in the biomorphs GP map means that a random mutation on a random genotype is more likely to give a specific simple phenotype than a specific complex one. Here, we test whether this continues to hold when we consider mutations for a fixed initial phenotype (i.e. whether there is simplicity bias in the ϕ_{pq} values). The data is shown in Fig L: for a fixed initial phenotype q, we compare the complexity of the ten phenotypes which are most likely to appear after mutations (i.e. with the highest ϕ_{pq} values), to the complexity of all phenotypes which can appear after mutations (i.e. with non-zero ϕ_{pq} values). This data indicates that, regardless of the initial phenotype q, phenotypic changes that happen with a high probability through mutations tend to be towards simpler phenotypes than phenotypic changes that occur with a lower probability. A caveat of this analysis is that we limit the set of initial phenotypes q to phenotypes, from which at least 100 different phenotypic changes are possible through mutations since the top-ten values are only relevant if there are $\gg 10$ non-zero ϕ_{pq} values.

E GP map properties with a fixed number of developmental stages

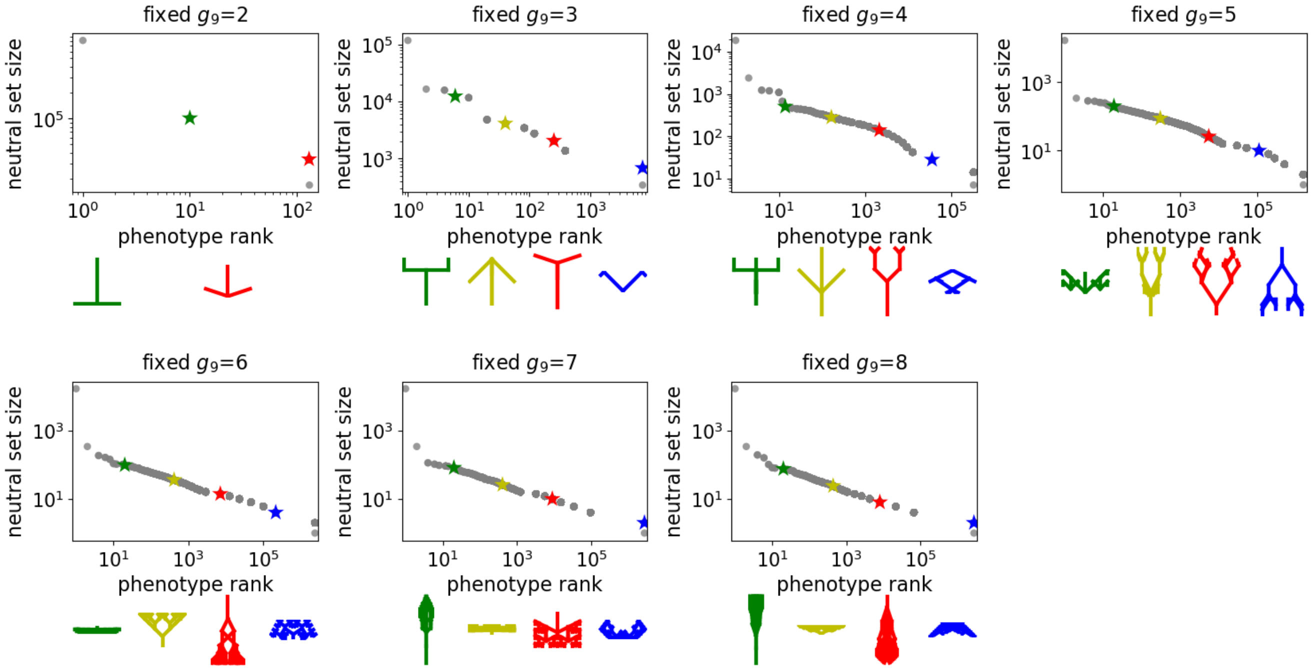

Figure M: ***Neutral set size vs rank for fixed values of*** g_9***:*** *each plot analyses the slice of the GP map that is defined by a fixed value of* g_9 *between* $g_9 = 2$ *and* $g_9 = 8$ *(indicated in the plot titles). In each case, the number of genotypes per phenotype (i.e. the neutral set size at fixed* g_9*) is computed for all phenotypes present in the given slice and plotted as a rank plot using our computational approach with the same parameters as in the main text. To illustrate, what kind of phenotypes are frequent/rare in each case, a few phenotypes are highlighted in each plot and drawn in corresponding colors underneath the plot.*

The limitations of the analytic model can be seen most clearly when we restrict the value of the ninth gene to a constant: in this case, the analytic model would predict that each phenotype goes through the same number of recursions in the developmental process and has the same number of unused genes and thus unconstrained sites. This would mean that all phenotypic characteristics - neutral set size, complexity, phenotype robustness, phenotype evolvability, phenotype mutation probabilities - are the same for all phenotypes. Here we test this prediction using our computational model for several choices of a fixed g_9. We find that:

- Phenotypic bias, i.e. differences in phenotypic frequencies, continue to exist (Fig M). One mechanism behind this bias can be understood by considering the 'simple vertical line' phenotype as an example, which is the most frequent phenotype for each value of g_9. This line phenotype is produced by a higher number of genotypes because it can be generated in many ways, by (overlapping) lines of arbitrary lengths, as long as all x-components are zero. By contrast, a more sophisticated shape imposes stricter constraints on the relative lengths and angles of the different vectors and is thus produced by fewer genotypes.

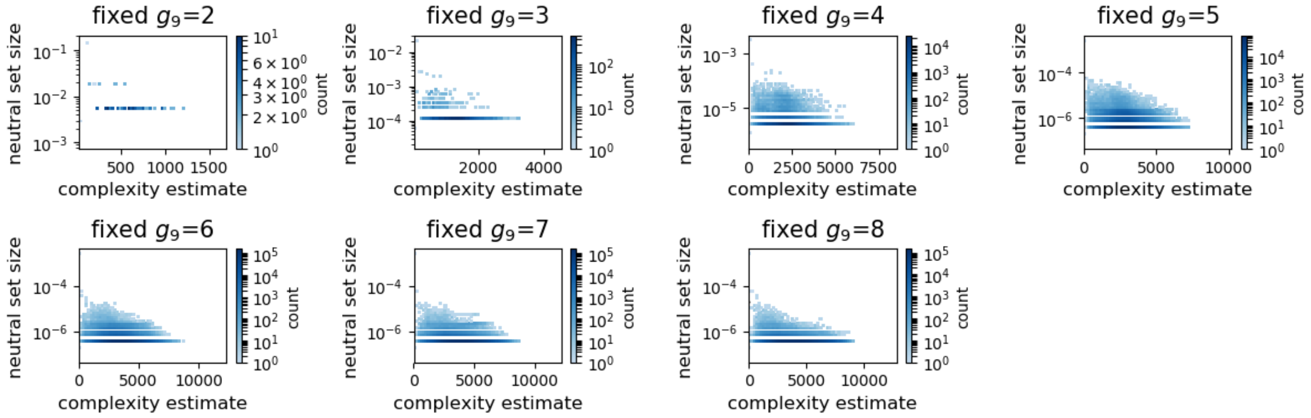

Figure N: ***Neutral set size vs estimated complexity, in a GP map with a fixed value of*** g_9***:*** *each plot analyses the slice of the GP map that is defined by a fixed value of* g_9 *between* $g_9 = 2$ *and* $g_9 = 8$ *(indicated in the plot titles). Otherwise, the plot follows Fig 4C in the main text. We find simplicity bias, despite constraining the value of* g_9*, and thus the number of developmental stages, to a constant.*

- Simplicity bias (Fig N & O): the most common phenotypes, such as the simple 'line' phenotype, tend to be simple.
- Genetic correlations, i.e. $\rho_p > f_p$, specifically an approximately log-linear relationship between phenotype robustness and frequency (Fig P): for example, two genotypes mapping to a single vertical line have zeros at all x-component-genes, and are thus more likely to be mutational neighbours than arbitrary genotypes.
- A negative relationship between robustness and evolvability on the genotypic level, and a (weak) positive one on the phenotypic level (Figs Q & R). The latter trend only appears for values of g_9 which give a high number of phenotypes, i.e. $g_9 \gtrapprox 4$.
- Mutation probabilities from an initial phenotype q (the line phenotype) to a new phenotype p are more likely for high-frequency target phenotypes p (Fig S). This trend only holds for $g_9 \gtrapprox 3$. Even then, it is not a perfect correlation, but this is in line with results for molecular GP maps [19], where the positive trend only serves as a first approximation.

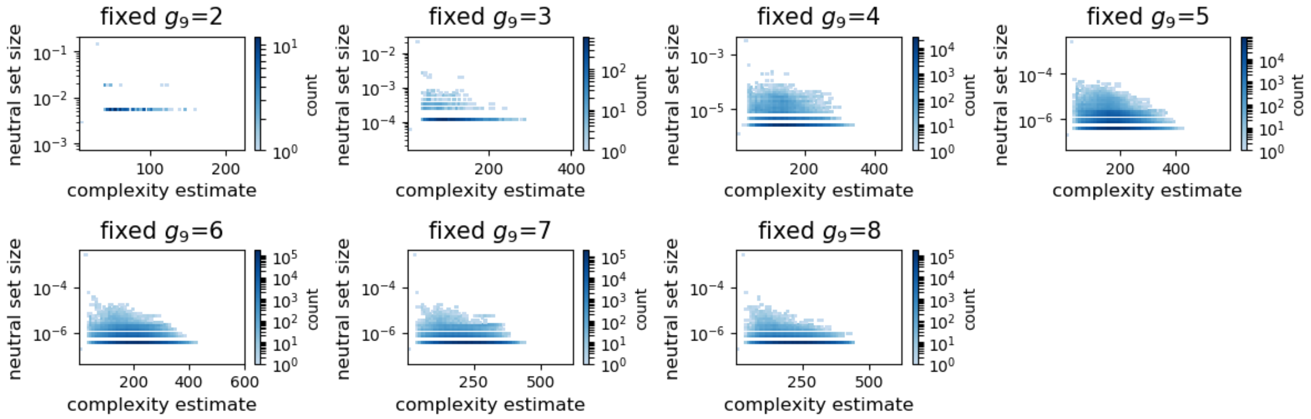

Figure O: ***Neutral set size vs estimated complexity, in a GP map with a fixed value of*** g_9***:*** *same as Fig N, but using an alternative complexity estimate based on Lempel-Ziv-based compression (as in section D.2). We find simplicity bias, despite constraining the value of* g_9*, and thus the number of developmental stages, to a constant.*

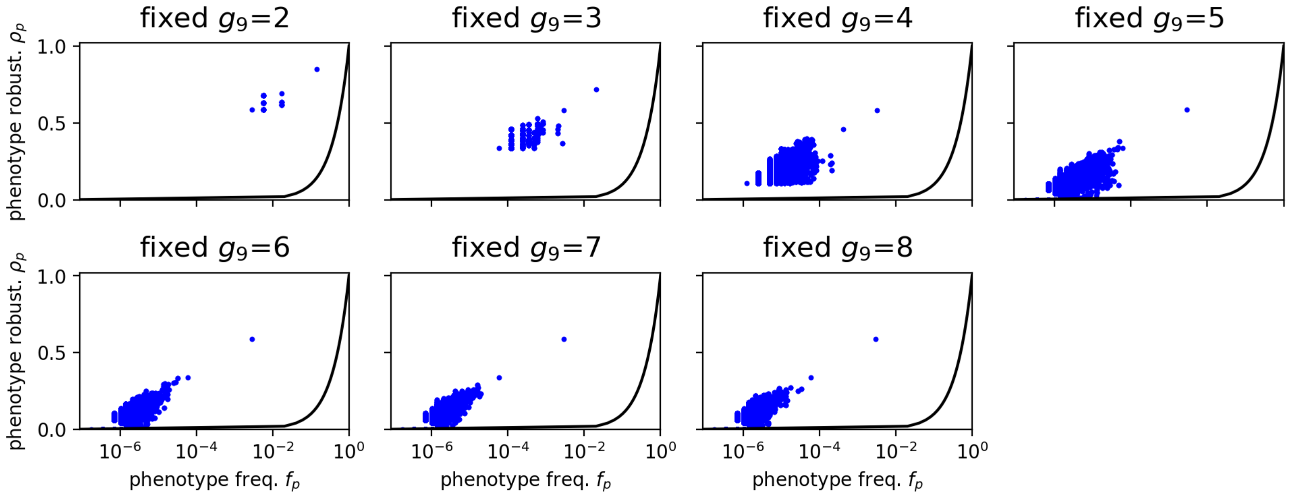

Figure P: ***Phenotypic robustness vs phenotypic frequency, in a GP map with a fixed value of*** g_9***:*** *each plot analyses the slice of the GP map that is defined by a fixed value of* g_9 *between* $g_9 = 2$ *and* $g_9 = 8$ *(indicated in the plot titles). Otherwise, the plot follows Fig 5B in the main text. As in the main text, we find* $\rho_p > f_p$ *with a roughly log-linear trend - this becomes clearer with an increasing number of phenotypes at higher fixed values of* g_9*.*

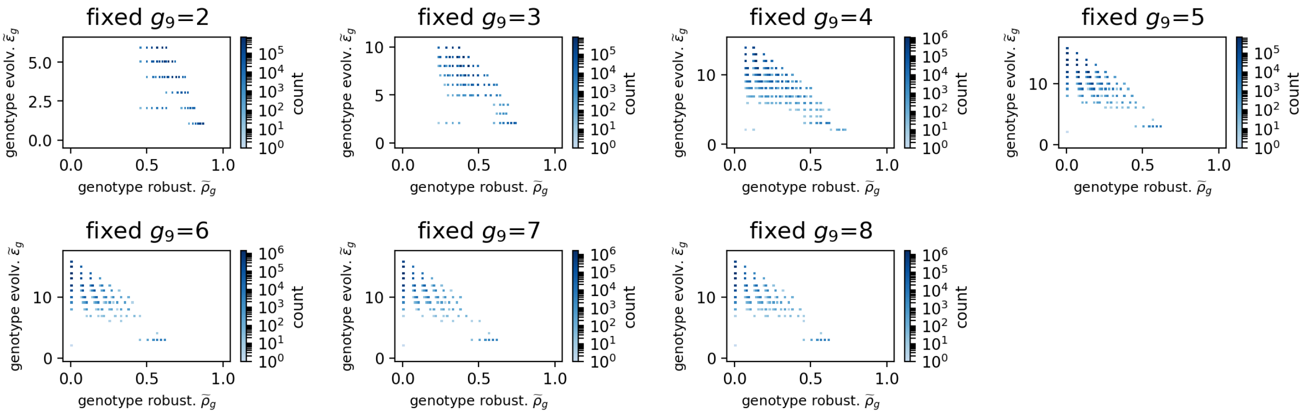

Figure Q: ***Genotypic evolvability vs genotype robustness, in a GP map with a fixed value of*** g_9***:*** *each plot analyses the slice of the GP map that is defined by a fixed value of* g_9 *between* $g_9 = 2$ *and* $g_9 = 8$ *(indicated in the plot titles). Otherwise, the plot follows Fig 5C in the main text. As in the main text, we find a trade-off.*

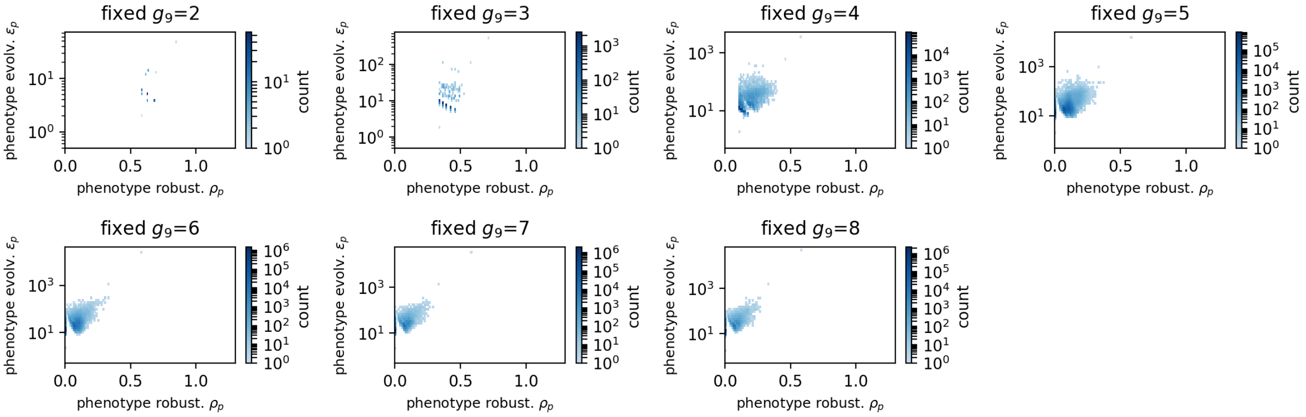

Figure R: ***Phenotypic evolvability vs phenotype robustness, in a GP map with a fixed value of*** g_9***:*** *each plot analyses the slice of the GP map that is defined by a fixed value of* g_9 *between* $g_9 = 2$ *and* $g_9 = 8$ *(indicated in the plot titles). Otherwise, the plot follows Fig 5D in the main text. As in the main text, we find a (weak) positive trend, even at fixed* g_9 *(at least for* $g_9 \gtrsim 4$*, where the number of phenotypes is high).*

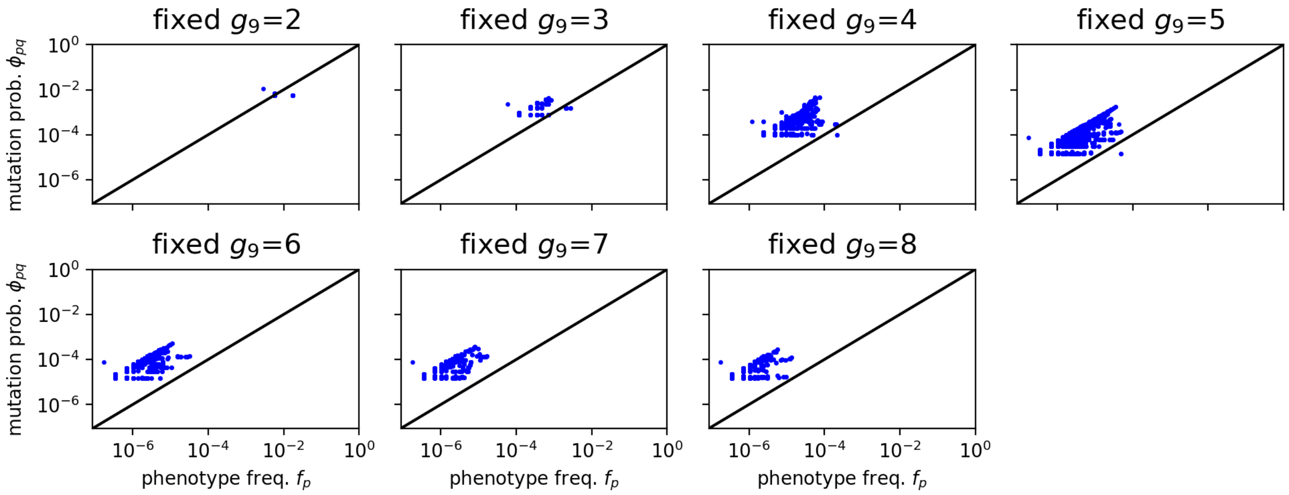

Figure S: ***Mutation probability ϕ_{pq} from an initial phenotype q to a new phenotype p vs phenotype frequency f_p, in a GP map with a fixed value of g_9:*** *each plot analyses the slice of the GP map that is defined by a fixed value of g_9 between $g_9 = 2$ and $g_9 = 8$ (indicated in the plot titles). The initial phenotype q is the 'vertical line' phenotype, which has the highest frequency for any g_9. Otherwise, the plot follows Fig 5E in the main text. As in the main text, we find that mutations to high-frequency phenotypes tend to be more likely (at least for $g_9 \gtrsim 3$).*

F Identifying an evolutionary path with the smallest number of phenotypic changes

In the main text, we illustrated how a line-shaped initial phenotype can be transformed into a specific insect-shaped phenotype in single point mutations, such that the number of phenotypic changes during the process is as small as possible. Here, we describe the computational approach we used to obtain this shortest series of phenotypic changes.

It is useful to reframe this optimization problem in terms of neutral components (NCs): a NC is a subset of the neutral set of p, which is defined [20] such that two genotypes are in the same NC if they can be connected by a series of neutral mutations. Therefore, it is possible to get from any genotype in a NC to any other genotype in the same NC during a period of neutral evolution, but any transition to another NC through mutations has to include phenotypic changes. NCs for a given GP map can be identified efficiently, for example by building on methods from ref [10].

Once we have enumerated all NCs, identifying the path with the smallest number of phenotypic changes is equivalent to finding the sequence of mutations with the smallest number of mutations that change the neutral component (NC) since two NCs that are connected by point mutations always correspond to different phenotypes, by definition. This allows us to solve our optimization problem by focusing on NCs and not individual genotypes. Thus, we created a unique ID for each NC in the GP map and evaluated, which NCs can be reached from a given NC through single point mutations. This defines a network, where each NC is a node and each edge indicates that there are point mutations connecting two NCs. Then we found the shortest path in this network using Dijkstra [21]'s algorithm. The resulting list of NCs gives the shortest list of NC transitions that have to be made in order to convert the initial phenotype into the target phenotype through point mutations. Then we simply mapped the NCs to the corresponding phenotypes to obtain the final figure.

G Two-peaked landscapes - beyond the first fixation

In the main text, we studied a scenario based on refs [8, 22], where a population with an initial phenotype p_0 evolved adaptively to one of two fitness peaks, p_1 or p_2. We reported which of these two phenotypes was the first to go into fixation, the mutationally more accessible phenotype p_1 or the phenotype with higher selective advantage p_2. Here, we investigate what happens after the first fixation event. Since there are no point mutations that can convert p_1 into p_2 directly (or vice versa), the only possible phenotypic changes are either back to the initial phenotype p_0, which is less fit, or directly to the other phenotype through a rare event involving multiple mutations at once. To investigate, if one of these phenotypic changes is likely to happen, we ran additional simulations that spanned 10^6 generations each and were not terminated with the first fixation event. This is about ten times longer than the typical number of generations until the first fixation event, which is typically between 10^4 and 10^5 (depending on the parameters s_1 and s_2). We ran 100 such simulations for each combination of parameters s_1 and s_2. We found no instances of direct fixations from p_1 to p_2, implying that the requirement for multiple specific mutations to coincide makes such transitions extremely unlikely for the mutation rates used in our simulations. Thus, the only new fixations that we observe after the first fixation event is a reversion to p_0, the phenotype with the lowest fitness. This

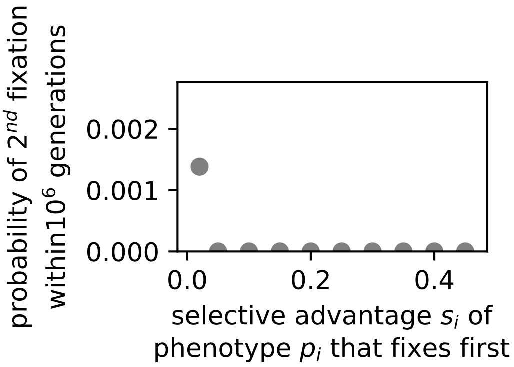

Figure T: ***Likelihood of reversion to fitness valley in the two-peaked landscape:*** *We simulate evolving populations in the two-peaked landscape described in the main text for* 10^6 *generations. The first fixation of one of the local maxima (p_1 or p_2) typically occurs after* $\lessapprox 10^5$ *generations. Here we investigate how likely the population is to experience a renewed fixation to the initial phenotype p_0 (the fitness valley). We plot this likelihood against the selective advantage s_i of the phenotype that was the first to fix. As might be expected, reversions to the fitness valley via drift are rare since we have strong selection with* $Ns_i \geq 10$. *The same parameters are used as in the main text, but the data is only based on 100 repetitions for each combination of the parameters s_1 and s_2 because of the higher computational cost of this analysis.*

only happens in rare cases ($< 0.5\%$ of cases) and only if the phenotype that fixes first only has a low selective advantage over p_0 (Fig. T).

H Selection for tree-like shapes

Here, we repeat the evolutionary simulation from Fig 6 in the main text, but with a slightly more complex fitness landscape inspired by one of the scenarios in Johnston et al. [16]: we assume that all tree-like phenotypes are equally fit (fitness $F = 1$) and all other phenotypes are completely unviable ($F = 0$). To obtain a reproducible and simple definition of what a tree-like shape is, we proceed as follows: we crop empty margins from the biomorph's grid representation and then consider the resulting cropped grid to be tree-like if there is a 'stem' in the lower part of the figure (i.e. the pixels on the lower fifth of the y-axis are filled in) and this stem is surrounded by some space (i.e. the pixels immediately to the right and left of the stem are not filled in). We also consider a single vertical line to be a tree-like phenotype.

The data is shown in Fig U: we find phenotypic bias over several orders of magnitude even among the more restricted set of tree-like phenotypes. This phenotypic bias is reflected in the evolutionary simulation: while selection confines the population to tree-like shapes, the phenotypic bias still plays an important role in determining *which* of the tree-like shapes appear more often in the evolving population. Here, some of the frequent shapes are actually observed more often than expected based on their phenotypic frequencies, which is likely due to selection for high robustness at this high mutation rate of $\mu = 0.1$ (the 'survival of the flattest' [23] effect).

The strong bias in the frequency of different tree-like shapes in the simulation is not surprising since transitions between different tree-like biomorphs are neutral in this scenario. However, it constitutes a simple

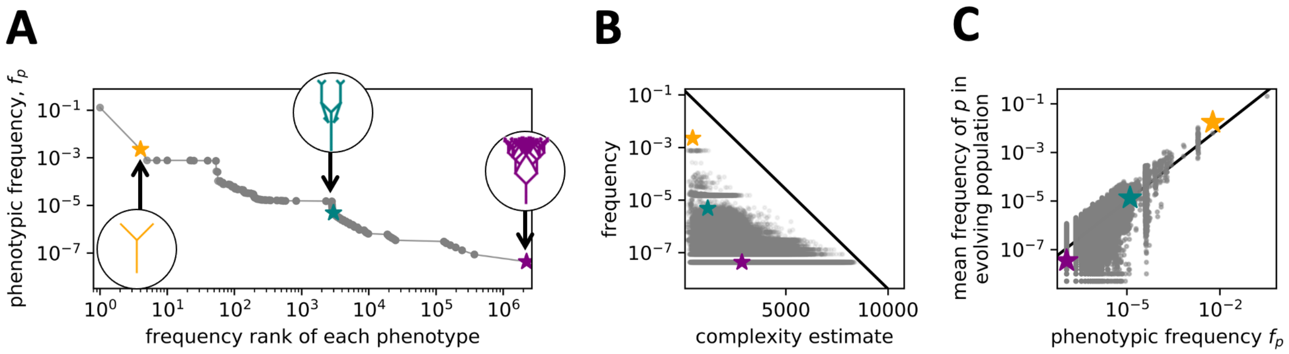

Figure U: ***Phenotypic bias towards simple shapes if the analysis is restricted to tree-like biomorphs:*** *The concepts of this plot are the same as in Fig 6 in the main text, but here only tree-like phenotypes have non-zero fitness in the evolutionary simulation: (A) Phenotype frequency vs rank for all tree-like phenotypes. Three phenotypes are selected from this plot: one with high frequency (yellow), one with medium frequency (teal), and one with low frequency (purple). We find bias even among tree-like phenotypes. (B) Phenotype frequency vs estimated complexity for all tree-like phenotypes, with the three selected phenotypes from (A) highlighted in color. We observe simplicity bias among the tree-like shapes. (C) As a simplified model of an evolutionary process, we assume that all tree-like phenotypes are equally fit (fitness $F = 1$) and all other phenotypes are completely unviable ($F = 0$). We model a population of 2000 individuals with a mutation rate of $\mu = 0.1$ for 10^5 generations. In this process, we record, how frequently we each of the selected shapes from (A) occurs. In (C), the normalized number of times each phenotype occurs in the population is plotted against a renormalized version of its phenotypic frequency (such that the frequencies of all tree-like shapes sum to one). We find that the phenotypic bias among the tree-like shape is reflected in their frequency in the evolving population.*

example, where there is selection on some features of the phenotype only, which may be a more realistic approximation under some conditions.

References

[1]R. Dawkins, *The Blind Watchmaker (appendix on evolvability added in 1991)* (W. Norton, 1986).

[2]R. Dawkins, "The evolution of evolvability", in *Artificial life : the proceedings of an interdisciplinary workshop on the synthesis and simulation of living systems, held September, 1987 in Los Alamos, New Mexico*, edited by C. G. Langton, Proceedings volume in the Santa Fe Institute studies in the sciences of complexity ; v. 6 (Addison-Wesley, Redwood City, Calif ; Wokingham, 1989), pp. 201–220.

[3]S. F. Greenbury and S. E. Ahnert, "The organization of biological sequences into constrained and unconstrained parts determines fundamental properties of genotype-phenotype maps", Journal of The Royal Society Interface **12**, 20150724 (2015).

[4]S. Manrubia and J. A. Cuesta, "Distribution of genotype network sizes in sequence-to-structure genotype - phenotype maps", Journal of The Royal Society Interface **14**, 20160976 (2017).

[5]M. Weiß and S. E. Ahnert, "Phenotypes can be robust and evolvable if mutations have non-local effects on sequence constraints", Journal of The Royal Society Interface **15**, 20170618 (2018).

[6]M. Weiß and S. E. Ahnert, “Using small samples to estimate neutral component size and robustness in the genotype–phenotype map of RNA secondary structure”, Journal of the Royal Society Interface **17**, 20190784 (2020).

[7]K. Dingle, C. Q. Camargo, and A. A. Louis, “Input-output maps are strongly biased towards simple outputs”, Nature Communications **9** (2018).

[8]S. Schaper and A. A. Louis, “The Arrival of the Frequent: How Bias in Genotype-Phenotype Maps Can Steer Populations to Local Optima”, PLoS ONE **9**, e86635 (2014).

[9]W. Arthur, “The concept of developmental reprogramming and the quest for an inclusive theory of evolutionary mechanisms”, Evolution & Development **2**, 49–57 (2000).

[10]W. Grüner, R. Giegerich, D. Strothmann, C. Reidys, J. Weber, I. L. Hofacker, P. F. Stadler, and P. Schuster, “Analysis of RNA sequence structure maps by exhaustive enumeration II. structures of neutral networks and shape space covering”, Monatshefte für Chemie/Chemical Monthly **127**, 375–389 (1996).

[11]E. Ferrada and A. Wagner, “A comparison of genotype-phenotype maps for RNA and proteins”, Biophysical journal **102**, 1916–1925 (2012).

[12]S. E. Ahnert, “Structural properties of genotype-phenotype maps”, Journal of The Royal Society Interface **14**, 20170275 (2017).

[13]P. Schuster, W. Fontana, P. F. Stadler, and I. L. Hofacker, “From sequences to shapes and back: a case study in RNA secondary structures”, Proceedings of the Royal Society of London. Series B: Biological Sciences **255**, 279–284 (1994).

[14]S. F. Greenbury, S. Schaper, S. E. Ahnert, and A. A. Louis, “Genetic correlations greatly increase mutational robustness and can both reduce and enhance evolvability”, PLOS Computational Biology **12**, e1004773 (2016).

[15]H. Zenil, S. Hernández-Orozco, N. A. Kiani, F. Soler-Toscano, A. Rueda-Toicen, and J. Tegnér, “A decomposition method for global evaluation of Shannon entropy and local estimations of algorithmic complexity”, Entropy **20** (2018).

[16]I. G. Johnston, K. Dingle, S. F. Greenbury, C. Q. Camargo, J. P. Doye, S. E. Ahnert, and A. A. Louis, “Symmetry and simplicity spontaneously emerge from the algorithmic nature of evolution”, Proceedings of the National Academy of Sciences **119**, e2113883119 (2022).

[17]P. K. Lehre and P. C. Haddow, “Phenotypic complexity and local variations in neutral degree”, Biosystems **87**, 233–242 (2007).

[18]A. H. Wright and C. L. Laue, “Evolving complexity is hard”, arXiv **2209.13013** (2022).

[19]N. S. Martin and S. E. Ahnert, “The boltzmann distributions of molecular structures predict likely changes through random mutations”, Biophysical Journal **122**, 4467–4475 (2023).

[20]S. Schaper, I. G. Johnston, and A. A. Louis, “Epistasis can lead to fragmented neutral spaces and contingency in evolution”, Proceedings of the Royal Society B: Biological Sciences **279**, 1777–1783 (2012).

[21]E. W. Dijkstra et al., “A note on two problems in connexion with graphs”, Numerische Mathematik **1**, 269–271 (1959).

[22]L. Y. Yampolsky and A. Stoltzfus, “Bias in the introduction of variation as an orienting factor in evolution”, Evolution and Development **3**, 73–83 (2001).

[23]C. O. Wilke, J. L. Wang, C. Ofria, R. E. Lenski, and C. Adami, "Evolution of digital organisms at high mutation rates leads to survival of the flattest", Nature **412**, 331–333 (2001).
